# Printing the Pathway Forward in Bone Metastatic Cancer Research: Applications of 3D Engineered Models and Bioprinted Scaffolds to Recapitulate the Bone–Tumor Niche

**DOI:** 10.3390/cancers13030507

**Published:** 2021-01-29

**Authors:** Anne M. Hughes, Alexus D. Kolb, Alison B. Shupp, Kristy M. Shine, Karen M. Bussard

**Affiliations:** 1 Department of Biomedical Engineering, Worcester Polytechnic Institute, Worcester, MA 01609, USA; amhughes@wpi.edu; 2 Department of Cancer Biology, Thomas Jefferson University, Philadelphia, PA 19107, USA; alexus.kolb@students.jefferson.edu (A.D.K.); alison.shupp@students.jefferson.edu (A.B.S.); 3 Health Design Lab, Jefferson Bioprinting Lab, Department of Emergency Medicine, Thomas Jefferson University, Philadelphia, PA 19107, USA

**Keywords:** bioprinting, breast cancer, metastasis, bone, tissue engineering, 3D modeling, tumor microenvironment, extracellular matrix

## Abstract

**Simple Summary:**

Breast cancer commonly migrates to the skeleton. Once patients have breast cancer in their bones, their quality of life is poor. Research has shown that the environment and structure of bone helps support metastatic breast cancer cell growth. Current models used in laboratories to study breast cancer that has migrated to bone do not include all the components of what happens in the human body. This is because technology in the past was limiting. Now, however, a new technology exists, called three-dimensional (3D) bioprinting, that “prints” living cells in geometries like the ones within the human body, to replicate a more realistic environment. In cancer research, 3D bioprinting allows researchers to more closely replicate events that occur when cancer cells migrate from a primary location to a secondary site, as well as model events in the environment of the secondary site. 3D bioprinting may also be used instead of animal models in some cases, and may speed up new drug discovery for cancer treatment.

**Abstract:**

Breast cancer commonly metastasizes to bone, resulting in osteolytic lesions and poor patient quality of life. The bone extracellular matrix (ECM) plays a critical role in cancer cell metastasis by means of the physical and biochemical cues it provides to support cellular crosstalk. Current two-dimensional in-vitro models lack the spatial and biochemical complexities of the native ECM and do not fully recapitulate crosstalk that occurs between the tumor and endogenous stromal cells. Engineered models such as bone-on-a-chip, extramedullary bone, and bioreactors are presently used to model cellular crosstalk and bone–tumor cell interactions, but fall short of providing a bone-biomimetic microenvironment. Three-dimensional bioprinting allows for the deposition of biocompatible materials and living cells in complex architectures, as well as provides a means to better replicate biological tissue niches in-vitro. In cancer research specifically, 3D constructs have been instrumental in seminal work modeling cancer cell dissemination to bone and bone–tumor cell crosstalk in the skeleton. Furthermore, the use of biocompatible materials, such as hydroxyapatite, allows for printing of bone-like microenvironments with the ability to be implanted and studied in in-vivo animal models. Moreover, the use of bioprinted models could drive the development of novel cancer therapies and drug delivery vehicles.

## 1. Introduction

Bone is a unique organ that provides, among other things, structural support and protection for the body. The bone, including the extracellular matrix (ECM), is continuously remodeled whereby components of the ECM are modified, secreted, or degraded [1]. The skeleton is composed of a variety of cells, including osteoblasts, osteocytes, osteoclasts, fibroblasts, endothelial cells, and mesenchymal stromal cells [2,3]. These cells are surrounded by organic and inorganic components such as type I collagen and apatite nanocrystals [2,3,4]. These organic and inorganic components constitute the bone matrix which is capable of providing biochemical and physiological cues that regulate bone modeling and mechanotransduction [3].

The skeleton is an attractive site for cancer cell dissemination and colonization due to its unique molecular and structural properties [5]. Although prognosis is excellent in patients with early non-invasive disease, 5-year mortality rates with treatment exceed 80% once cancer has invaded the bone [6]. Normal bone processes, such as bone remodeling, produce a plethora of growth factors and cytokines that are strong chemoattractants for metastatic cancer cells [7,8,9,10,11]. Once in the bone, metastatic cancer cells hijack the endogenous stromal cells in the bone microenvironment, including osteoblasts and osteoclasts, to remodel the bone matrix, leading to a disorganized basement membrane and disrupted cellular crosstalk, which ultimately promote tumor progression [12,13,14]. Thus, understanding the interactions between the ECM and metastatic cancer cells is crucial in order to both regulate and prevent metastatic cancer cell growth in bone. However, the study of cancer cell dissemination, seeding, colonization, and survival in bone is complicated by the vast structural and physiological complexities of the organ. Most model systems currently available only account for select aspects of the niche; thus, a combination of techniques is frequently utilized to wholly recapitulate events that occur during bone–tumor crosstalk.

Two-dimensional (2D) in-vitro cell culture environments have arisen as important platforms for the study of cancer cell proliferation, migration, and invasion as well as cancer cell response to therapeutic treatment. Although simple and low cost, the planar geometry and non-physiologic composition (i.e., tissue culture plastic) of 2D cultures can induce variable cellular morphologies and aberrant gene expression [15,16,17] that impact the behavior and cellular crosstalk of endogenous bone and tumor cells [15,18]. Moreover, mechanical cues translated by the microenvironment are absent [3]. Importantly, Bissell and colleagues discovered that normal breast epithelial cells required interactions with the basement membrane, as present in three-dimensional (3D) cultures, to display and maintain their normal phenotype. Otherwise, the normal breast epithelial cells behaved similarly to tumor cells when grown in 2D cultures [19]. Thus, such models inadequately represent the spatial, biochemical, and mechanical complexities of the native 3D tumor microenvironment, severely limiting their interpretation in the study of metastatic disease to the bone.

Recognizing these shortcomings, investigators are now developing 3D bone microenvironments such as bioreactors [20,21,22,23,24,25,26], scaffolds [27,28], extramedullary bone [29,30], and bone-on-a-chip [31,32] models for metastatic cancer research. Such novel systems allow for more relevant material composition and spatial relationships among cells and, in some cases, provide similar mechanical forces. However, these systems face individual challenges including reproducibility, scale, and ability to manipulate matrix biochemical composition and structural integrity, and can be difficult to seed with cells for culture [33]. Thus, a gold standard model for the study of cancer metastasis to bone remains elusive.

3D bioprinting, an evolving technology, may help to facilitate such research. A novel biofabrication technique that yields reproducible, biomimetic 3D environments composed of cells and native biomaterials, 3D printing offers distinct advantages over prior 2D and 3D cell culture models. ECM composition and matrix geometry and stiffness, as well as cell density and position, are all controllable parameters in bioprinted 3D scaffolds, supporting cellular proliferation, migration, phenotype development, and matrix and biochemical factor production [16]. 3D constructs can achieve biophysical characteristics similar to the native tissue microenvironments, allowing for the study of cancer progression and tumorigenesis [34]. Thus, 3D bioprinting may provide a more robust solution, yielding biomimetic microenvironments for the study of cell–cell and cell–matrix interactions at the forefront of cancer research.

In this review, we focus on cancer metastases to bone and provide an overview of current 2D and 3D in-vitro culture model systems, as well as 3D in-vivo and ex-vivo model systems to study cancer metastases to bone. We review the applications and limitations of current technologies, which include the inability of current models to wholly represent the spatial, biochemical, and mechanical complexities of the native 3D tumor microenvironment, causing limited interpretation of results generated in these systems. We next discuss an emerging technology called 3D bioprinting. 3D bioprinted bone-mimetic microenvironments offer distinct advantages over current 2D and 3D technologies, including reproducibility and the ability to incorporate native biophysical properties of the ECM, including cellular composition, stiffness, and geometry. As a result of these and other advantages, 3D bioprinting has the potential to elevate into the next generation current models in cancer research that mimic the bone–tumor niche.

## 2. Gross Anatomy of Bone and Bone Physiology

Bone is a metabolically active and dynamic organ that supports many body functions, including regulation of calcium blood levels, providing structural support, and protecting internal organs. To accomplish these and other functions, bone is composed of three main cell types: osteoblasts, osteoclasts, and osteocytes. Osteoblasts, the cells that synthesize new osteoid matrix, account for 4-6% of total cells in bone. Osteoblasts are derived from mesenchymal stromal cells present in the bone marrow [35]. After stimulation with bone morphogenetic proteins and growth factors, mesenchymal stromal cells proliferate to form pre-osteoblasts, which then differentiate into mature osteoblasts capable of matrix synthesis and deposition [36]. Newly formed bone is composed of type I collagen and non-collagenous proteins (proteoglycans and other extracellular matrix proteins) (~22%), hydroxyapatite (~70%), and water (~8% by weight) [35,37,38].

Osteoclasts, on the other hand, are the cells responsible for bone resorption. Osteoclasts account for 1–4% of total cells in the bone, and are derived from bone monocytes [39,40]. Bone marrow monocytes (i.e., precursor osteoclasts) express Receptor Activator for Nuclear factor Kappa beta (NF-κB)(RANK) on their cell surface, which binds to Receptor Activator for Nuclear factor Kappa beta Ligand (RANK-L) on osteoblasts to elicit osteoclastogenesis [41]. Several single-nucleated osteoclasts then fuse together to form one mature, multi-nucleated bone-resorbing osteoclast [41]. Upon subsequent activation, osteoclasts carry out a complex degradation process to secrete matrix degradation enzymes and resorb bone. This process exposes bones’ organic matrix via degradation by cathepsin K and additional cysteine proteinases, lysozymal enzymes, phosphatases, and matrix metalloproteinases at the osteoclast–bone interface [41,42,43,44,45,46]. As bone is resorbed, growth factors stored in the bone matrix, including transforming growth factor-beta (TGF-B), insulin growth factor (IGF), and bone morphogenetic proteins (BMPs), are released into the microenvironment [47].

Osteocytes help to regulate the activities of osteoblasts and osteoclasts via mechanotransduction, and represent ~90–95% of cells in the bone [48,49]. Osteocytes are formed when osteoblasts become terminally differentiated and embedded in the bone matrix. Osteocytes reside in small cavities, called lacunae, which transmit signals to bone cells, including osteoblasts and osteoclasts, via tiny channels called canaliculi [49]. These signals regulate nutrient exchange in bone, as well as bone remodeling and sensation of mechanical stimuli [3,50,51,52,53]. As a result of their ability to sense mechanical load in the bone, osteocytes have earned the nickname the “mechanosensors of bone” [54,55,56].

## 3. Bone Remodeling during Disease

Bone resorption and deposition are held in a tightly regulated, homeostatic balance in which there is no net bone loss or gain. However, this balance is upset in pathological conditions, including infection (osteomyelitis), osteoarthritis, and bone metastatic cancers [57,58,59,60]. In each of these conditions, and especially in osteolytic disease related to cancer, osteoclasts are overstimulated to degrade bone. Osteoblasts do not deposit new bone; thus resulting in net bone loss [61]. In brief, osteomyelitis is a severe bone infection, caused by the bacterium *Staphylococcus aureus*, which can be fatal [62,63]. Osteomyelitis typically occurs from chronic inflammation as a result of osseointegrated implants (e.g., dental implants or femoral implants (i.e., artificial hip)) [64,65,66]. Osteomyelitis involves the internalization of *S. aureus* by osteoblasts, permitting *S. aureus* to evade immune detection and cause sustained inflammation [67]. Upon internalization of *S. aureus*, osteoblasts increase their production of inflammatory cytokines, including interleukin-6 (IL-6), monocyte chemoattractant protein-1 (MCP-1), regulated on activation normal T cell expressed and secreted (RANTES), and macrophage inflammatory protein-1 alpha (MIP-1 alpha), as well as factors that stimulate osteoclastogenesis, including granulocyte-colony stimulating factor (G-CSF), RANK-L, and interleukin-8 (IL-8), among others [68,69,70,71,72,73,74]. Sustained infection leads to a reduction in osteoblast proliferation and eventual osteoblast death. Subsequently, bone is resorbed at an increased rate by osteoclasts, leading to sustained bone loss [74]. Osteoarthritis is another bone disease characterized by chronic inflammation that impacts osteoblast function. In osteoarthritis, osteoblasts overexpress inflammatory cytokines, including IL-8, IL-6, vascular endothelial growth factor (VEGF), and prostaglandin E2 (PGE2), among others [59,60,75]. This results in a reduction of osteoblast bone deposition, and an imbalance in bone remodeling that results in sustained bone resorption. [76,77]. Interestingly, many of the same cytokines and inflammatory factors overexpressed by osteoblasts during osteomyelitis and osteoarthritis have also been known to be overexpressed during bone invasion by metastatic cancer cells, including breast, prostate, lung, and multiple myeloma [7,26,78,79,80,81,82].

## 4. Bone Is a Favored Site of Cancer Metastasis

Bone is a preferential site of cancer metastasis [8,9]. In an attempt to explain directional tropism of disseminated cancer cells for specific organs in the body, Stephen Paget made the following statement in 1889: “When a plant goes to seed, its seeds are carried in all directions; but they can only live and grow if they fall on congenial soil [83]”. As suggested by Paget, disseminated tumor cells are the “seeds” and the bone niche, rich in growth factors, neovascularization factors, and cytokines, is the “congenial soil” necessary for cancer cell growth [84]. Paget’s “seed and soil” hypothesis also explains the preferential metastasis of breast, prostate, lung, and multiple myeloma disseminated cancer cells to the bone. Furthermore, mounting evidence has implicated the cells of the bone responsible for remodeling, the osteoblasts and osteoclasts, as key players in bone metastatic cancer cell progression, including cancer cell homing to and seeding in bone, dormancy, cancer cell re-activation, and contribution to macrometastatic lesion growth [85,86].

Once shed from a primary tumor, disseminated tumor cells enter the circulation, ultimately traveling to large blood vessels found in the bone called vascular sinusoids. Blood flow within the sinusoids is sluggish, allowing for normal movement of immune, hematopoietic, and lymphoid cells into and out of the bone. This slow blood flow also enables cancer cells to easily invade bone simply due to the normal physiology [8,87]. The vascular sinusoids are the main entry point for disseminated tumor cells into the bones, with the majority of metastases occurring at the ends of long bones, including the femur, where there is a high rate of metabolic activity due to bone turnover by the osteoblasts and osteoclasts.

### 4.1. Breast Cancer Cells Preferentially Metastasize to Bone

Approximately 20-30% of breast cancer patients will develop metastatic lesions, with ~15% of those being bone metastases [88,89]. Approximately 50% of these metastases will involve bone as a primary secondary tissue, with nearly 80% being secondary or recurring metastatic sites [82,90,91,92]. Once breast cancer invades bone, the relative 5-year survival rate falls to less than 10% [90]. Lesions in the bone that form as a result of bone metastatic breast cancer are predominantly osteolytic in nature, however, some lesions can also be mixed lytic and blastic [10,87,91,92,93]. During the formation of osteolytic lesions, osteoclasts are constitutively activated to resorb bone, and osteoblasts do not deposit new bone, resulting in sustained bone degradation. This phenomenon has been described as the “vicious cycle” of breast cancer metastases to bone [9,25,94]. Occurring during advanced tumor progression, metastatic breast cancer cells produce parathyroid hormone-related protein (PTHrP), which stimulates osteoblasts to produce increased amounts of RANK-L. Osteoblasts that overexpress RANK-L bind to the receptor RANK on osteoclast precursors, stimulating osteoclastogenesis and increased bone resorption. Growth factors stored in bone are released, including TGF-beta, which are used by cancer cells to produce additional PTHrP [9,94], ultimately leading to sustained bone resorption and osteolytic lesion formation. Bone pain, fractures, hypercalcemia, and spinal cord compression subsequently occur [9,95,96]. Clinically, patients with osteolytic lesions are treated with drugs such as bisphosphonates that block the activity of osteoclasts, thus reducing bone degradation [97,98,99,100]. However, these drugs are not curative for the lesions already present; currently, no therapeutics are available that have the sole purpose to directly stimulate new bone deposition by osteoblasts.

### 4.2. Multiple Myeloma Metastases to the Bone

Multiple myeloma is another cancer that preferentially metastasizes to bone [101,102]. Approximately 70% of patients presenting with multiple myeloma have bone metastases upon diagnosis. Over time, over 90% of patients with multiple myeloma will develop bone metastases during the course of their disease [103]. The vast majority of these metastases will be osteolytic in nature, with bone being resorbed at a rate greater than it is deposited. Multiple studies by Roodman and colleagues have determined this is due to metastatic multiple myeloma cell suppression of osteoblast differentiation via a variety of factors [104,105,106,107,108,109,110]. At present, two main treatment strategies are being evaluated either in pre-clinical or clinical trials for the treatment of osteolytic metastases due to bone metastatic multiple myeloma. First, in pre-clinical models, treatment with an antibody to sclerostin, a Wnt/beta-catenin antagonist, reduced osteolytic lesion formation by 60% compared to wild-type mice. In addition, in tumor-bearing mice with a genetic deletion of sclerostin, there was a 50% increase in trabecular bone volume as well as an increase in the number of osteoblasts compared to tumor-bearing, wild-type mice [106]. Furthermore, as part of a phase I/II clinical trial, an inhibitor of DKK1, which is also involved in the Wnt signaling pathway, was found to increase osteoblast differentiation and calcium deposition in co-cultures of osteoblasts plus multiple myeloma cells in-vitro. That same DKK inhibitor increased both trabecular bone volume and Wnt/beta-catenin signaling (crucial for osteoblast differentiation) in tumor-bearing mice when compared to placebo [111]. Taken together, these results suggest that the inhibition of sclerostin and/or inhibitors of DKK1 may be promising tools to promote osteoblast activity, leading to increased subsequent bone deposition in multiple myeloma bone disease.

### 4.3. Prostate Cancer Colonization of the Skeleton

Prostate cancer has a predilection for bone metastases. Greater than 80% of patients with late-stage prostate cancer will develop bone metastases [9]. As opposed to other cancers with strong tropism for the bone, the vast majority of metastatic lesions that occur as a result of prostate cancer are osteoblastic in nature in which there is an excess of bone deposition [112]. This is due to prostate cancer cells preferentially homing to the ends of long bones where osteoblasts predominantly reside, leading to increases in both osteoblast and prostate cancer cell proliferation [80,113]. Interestingly, newly formed bone is disorganized, typically with low density, and is weak in strength [114,115]. This is due to a malalignment of osteoblasts along a collagen matrix, leading to the production of bone with a spongy structure as opposed to a hard and compact lamellar structure [116,117]. There are some reports, however, of bone metastatic prostate cancer lesions being mixed or osteolytic [118,119].

Similar to the “vicious cycle” of breast cancer metastases to the bone, bone metastatic prostate cancer cells are involved in a cycle that promotes excessive bone deposition by osteoblasts and continued proliferation of metastatic prostate cancer cells. Prostate cancer cells in the bone produce growth factors, including TGF-beta and IGF-1, as well as bone morphogenetic proteins that drive osteoblasts to deposit new bone [81,95,120,121]. In turn, bone resorption by osteoclasts is suppressed, leading to sustained net bone deposition [94,122]. In addition, activated osteoblasts overexpress inflammatory cytokines, including IL-6, IL-8, MCP-1, and VEGF, that fuel metastatic prostate cancer cell growth in bone [123]. A number of clinical trials are underway to treat osteoblastic lesions associated with bone metastatic prostate cancer. In a phase I/II clinical trial, atrasentan, an antagonist to the endothelin receptor A, reduced bone pain, slowed metastatic prostate tumor growth, and resulted in a significant decrease in median time to disease progression when compared to placebo [124,125]. In addition to atrasentan, Radium-223, an alpha particle emitter that preferentially accumulates at sites rich in osteoblast activity, has proven to be a promising therapeutic for patients with bone metastatic prostate cancer. In pre-clinical models, tumor-bearing mice exposed to Radium-223 had reduced bone growth, and preserved bone volume and architecture [126].

### 4.4. Lung Cancer Metastasizes to the Skeleton

Lung cancer is another cancer that has a tropism for the bone, although less so than breast cancer, prostate cancer, and multiple myeloma; lung cancer metastasizes to the bone approximately 34.3% of the time [127]. While exact mechanisms for preferential metastasis of lung cancer to bone are unclear, recent data implicate growth factors including TGF-beta [128], cytokines and chemokines such as stromal-derived factor-1 (SDF-1) and CXCR4 [129], and matrix metalloproteinases [129] that help drive lung cancer metastases to bone. Of the many types of lung cancer, small cell lung cancer and non-small cell lung cancer present mainly with osteolytic bone metastases in the spine and ribs [130,131,132]. Additional data suggest that lesions associated with bone metastatic lung cancer can also be mixed osteolytic and osteoblastic [130,131,132]. Clinically, markers of bone turnover, including osteopontin, collagen type I, and bone sialoprotein, can be used as biomarkers for the evaluation of bone metastatic lung cancer progression in human patients [133,134,135,136,137,138,139,140]. Osteopontin in particular has been associated with an aggressive lung cancer phenotype whereby increased osteopontin expression promotes lung cancer cell migration and invasion in bone via increased epithelial-to-mesenchymal transition [141,142].

## 5. Models to Investigate Tumor-Bone Cell Interactions

### 5.1. Model Systems for Early Cancer Cell Dissemination to Bone

Upon dissemination to bone, a cancer cell will either proliferate to form a metastatic lesion, or the cancer cell may enter a proliferatively quiescent or dormant state [143,144,145]. Sometimes also named “metastatic latency” [85], cellular dormancy can be defined as either proliferative arrest or a state of balanced proliferation and apoptosis where there is no net growth. In many situations, this is an adaptive response to microenvironmental stress at a secondary site whereby disseminated tumor cells must respond to unknown signals from the niche in order to survive [143]. Over time, dormant cancer cells become reawakened, leading to cancer cell proliferation and the formation of macrometastases in bone. At present, there is no model available that fully recapitulates all of the steps of early human cancer cell dissemination to bone. While a number of pre-clinical models are available that recapitulate specific steps of established disease, efforts to specifically study earlier events in bone metastatic cancer progression, especially early dissemination to bone, initial cancer cell seeding of bone, and events associated with metastatic latency, have been hampered by a lack of mouse and ex-vivo model systems due to substantial technical limitations [146]. Most importantly, there are no immune-competent mouse models capable of recapitulating all steps of the metastatic cascade available to investigate the role of the immune system during disease progression. As a result, knowledge of the early events of disease progression is severely limited.

Presently, only a handful of studies have attempted to investigate early steps of cancer metastasis to bone. Of those, osteoblasts in the endosteal bone niche have been identified as key mediators of cancer cell seeding. Scimeca et al. assessed human tissue samples of benign breast lesions, breast-infiltrating carcinomas, and bone metastatic breast cancer lesions [147]. The authors found a large number of breast cancer cells that underwent epithelial-to-mesenchymal transition (EMT) in the infiltrating carcinomas, which corresponded to a large number of breast–osteoblast-like cells that were positive for RANKL expression as well as the vitamin D receptor. These breast–osteoblast-like cells were also discovered in matched bone metastases [147]. These data suggest that osteoblasts and osteoblast-like cells may be used as an early predictor of bone metastatic breast cancer. Bodenstine et al. attempted to describe mechanisms that regulate osteoblast populations during the early stages of bone metastatic breast cancer [148]. An intratibial injection model was used to introduce osteoblasts plus bone-tropic breast cancer cells into the tibia to assess tumor growth. In mice injected with cancer cells alone (control), tumors that formed were smaller in size and remained contained in the bone cavity. By comparison, breast cancer cells co-injected with osteoblasts formed large palpable tumors nearly double the size of those formed with cancer cells injected alone, that migrated into the extra-osseous space [148]. These data suggest intracellular crosstalk between osteoblasts and bone metastatic cancer cells is important in cancer cell progression. However, while the mouse model used may not be fully representative of early-stage events, it may still help shed light on crosstalk that occurs between osteoblasts and metastatic breast cancer cells in bone.

Our laboratory additionally utilized a mouse model of intratibial injection to study interactions between osteoblasts and bone-tropic breast cancer cells in early disease progression. We identified a subset of osteoblasts present in the bone–tumor niche that regulate breast cancer progression in bone by producing inhibitory factors that reduce breast cancer cell growth [85]. We called this subset “educated” osteoblasts (EOs). Remarkably, tumors formed in mouse bones in the presence of EOs were smaller and grew at a slower rate than tumors composed of naive osteoblasts. We identified candidate proteins that distinguish “educated” osteoblasts (EOs) from naïve osteoblasts both in-vivo and ex-vivo. Using these markers, we interrogated human patient samples of bone metastatic breast cancer and identified EOs in patients with estrogen receptor positive (ER+) breast cancer [85,93]. Moreover, we demonstrated that exposure to EO conditioned medium reduced breast cancer cell proliferation and led to a reduction in the number of cells in the S phase of the cell cycle of both triple negative (TN) and ER+ breast cancer cells in-vitro. Furthermore, direct co-culture with EOs increased TN and ER+ breast cancer expression of p21 compared to cultures with naïve osteoblasts [85]. Similar to our findings in bone metastatic breast cancer, Lawson et al. found that osteoblasts and osteoclasts in the bone were capable of promoting (osteoblasts) or reawakening (osteoclasts) multiple myeloma cells from proliferative quiescence using an intravenous injection syngeneic mouse model as well as in-vitro analyses [86]. The authors used intravital imaging to track single multiple myeloma cells as they entered the bone niche and found that when fluorescently labeled multiple myeloma cells directly engaged with osteoblasts, myeloma cell growth was suppressed [86]. The authors recapitulated this in-vitro, whereby exposure to the conditioned medium of or co-culture with murine osteoblasts increased the number of dormant multiple myeloma cells. Conversely, when exposed to osteoclast-precursor conditioned media, multiple myeloma cells exhibited increased proliferation and growth [86]. Interestingly, daily injection of soluble RANK-L into tumor-bearing mice resulted in a reduction of dormant multiple myeloma cells and subsequent increase in osteoclastogenesis and bone resorption. In two additional studies, osteoblast expression of TGF-beta and Gas6 [149], as well as BMP7 [150], maintained bone metastatic prostate cancer cells in a dormant state, whereas reduction of those factors promoted prostate cancer cell growth. Thus, these data suggest engagement with osteoblasts either via direct or indirect means is key in the regulation of bone metastatic cancer cell progression.

### 5.2. Models to Investigate Late-Stage Metastatic Cancer Cell Invasion, Colonization, and Survival in Bone

Currently, there are no models available, in-vitro, ex-vivo, in-vivo, or otherwise, that fully recapitulate all the steps of the human bone metastatic cascade. This is mainly due to extensive technical hurdles and limitations, limited available human cell lines, limited immune-competent models, and sustaining long-term cell growth of primary human stromal cells [151,152,153,154]. As a result, multiple experimental models are used to investigate specific aspects of the metastatic cascade, with each mainly designed to investigate a specific stage of metastasis. Often, a combination of model systems is used to answer a given experimental question. For example, we and others have used complimentary approaches to investigate breast cancer metastasis in bone, including, but not limited to, multiple human cell lines [155,156], extended culture bioreactors [156], poly-ether-urethane foam scaffolds [157], biocompatible printed scaffolds [33], and novel engineered organotypic models [154].

#### 5.2.1. In-Vivo Models of Cancer Metastasis to Bone

In-vivo mouse models are a common pre-clinical assay to study a variety of steps of the bone metastatic cascade. Syngeneic mouse models refer to murine cancer cells injected into mice (i.e., same genetic background as the host), whereas xenograft models describe a host that is genetically distinct from the cancer cells inoculated (i.e., human cancer cells inoculated into mice). Each model has its own advantages and disadvantages: syngeneic models permit the analysis of an immune-competent system, as well as allow investigation between inoculated cells and endogenous stromal cells within the microenvironment [158]. However, there are proteins and genes that are not analogous between humans and mice (e.g., IL-8 (human) and MIP-2 (murine); GRO-alpha (human) and KC (murine)). Therefore, mechanisms that may be identified via a syngeneic mouse model may not directly translate to humans [158]. Xenograft models permit the study of human cancer cells in an immune-compromised host such that mechanisms specific to human cancer cell progression may be studied. In many cases, human and mouse cells are capable of uninhibited crosstalk [158,159]. However, xenograft models do not permit the study of the immune system with cancer progression.

Syngeneic and xenograft models may further be broken down based on the route of injection, specifically via the orthotopic, intracardiac, or intratibial route [160]. In an orthotopic model, cancer cells or fragments are implanted into the same anatomic location from which the cancer originated; i.e., breast cancer cells inoculated into the mammary gland or prostate cancer cells inoculated into the prostate of a mouse. While orthotopic models are very beneficial in studying primary cancer progression, many times, a primary tumor may out-pace the growth of any metastases that may arise. In these cases, primary tumors may be removed upon reaching a certain size, subsequently allowing metastases in secondary sites to progress. Depending on the growth of the inoculated cancer cells, progression to metastasis in orthotopic models may be prolonged, requiring multiple months of study [161]. Experimental metastasis models permit the investigation of steps involving the trafficking of cancer cells to secondary sites, as well as investigation of late-stage metastases. Intracardiac injections recapitulate cancer cell trafficking and homing to secondary organs via the vasculature due to the cancer cells being directly injected into the left ventricle of the heart, which bypasses pulmonary circulation [160]. Several laboratories have developed bone-tropic cell lines utilizing intracardiac mouse models, which more closely mimic bone metastases in humans [162,163]. In the majority of cases, intracardiac injections are carried out in a xenograft model due to fluorescent labeling of inoculated cancer cells for tracking and retrieval for ex-vivo analysis. Finally, intratibial injections are a model of established disease whereby cancer cells are directly injected into the trabecular bone of the tibia of mice. Tumors rapidly form, permitting the study of the bone microenvironment during late-stage disease [160].

#### 5.2.2. Three-Dimensional Tissue-Engineered Models to Study Cancer–Bone Interactions during Disease Progression

Current models to investigate complex cancer cell–bone interactions include tissue-engineered bone constructs (TEBCs), bone-on-a-chip (BC) bone tissue models, bioreactors for continuous culture, three-dimensional printed bone matrix scaffolds, hydrogels, and extramedullary bone models [164,165,166]. While these models replicate physiological systems better than standard two-dimensional cell culture due to their biophysical properties, all of these models fall short of being able to fully recapitulate the complexity and cellular crosstalk that occurs in the bone–tumor microenvironment. As a result, many investigators use a combination of 2D cell culture, 3D tissue-engineered models, and in-vivo models to investigate specific steps of the metastatic cascade.

##### Hydrogel Tissue Constructs

Hydrogels can be used in both two- and three-dimensional formats and are available as either natural (i.e., collagen) or synthetic (i.e., polyacrylamide) materials [167,168,169,170]. Of the types, three-dimensional hydrogels best mimic native bone, recapitulating tissue stiffness and bone’s elastic modulus, which most closely represent the mechanotransductive properties of the skeleton [164]. Furthermore, three-dimensional hydrogels permit the embedding of cells to be studied, which facilitate the study of cellular migration and invasion through a matrix. This also enables seeded cells to resemble their normal morphology in-vivo, thus allowing for better representation of physiologic events that occur during cell–cell and cell–matrix contact. While beneficial, the increased complexities involved in the use of cell-containing three-dimensional hydrogels (e.g., viability-maintaining fabrication techniques, crosslinking, and nutrient diffusion) may increase resource needs relative to more simple two-dimensional models (e.g., gel-coated polystyrene) as well as create technically challenging analysis via full thickness penetration of the hydrogel for imaging [167,170]. Because of these limitations, two-dimensional hydrogels are beneficial for easier cell observation and analysis, as well as the ease of manipulation of microenvironmental conditions. Two-dimensional hydrogels are also well established. A major disadvantage of two-dimensional hydrogels, though, is the inability to permit three-dimensional tissue structure, including flattened cell morphology, forced cell polarity (i.e., cells seeded on top of a hydrogel as opposed to within), and a high matrix stiffness due to the conformation of the hydrogel being flat [164].

##### Bone-Like Scaffolds

Bone-like scaffolds are one 3D model system frequently used to model the bone microenvironment [107,117]. These models are capable of recapitulating the rigid extracellular matrix of bone. Polymers such as poly-(ϵ-caprolactone) (PCL) [114,115], polylactic acid (PLA), or PCL/PLA blends [116] have been used to print 3D scaffold structures onto which cells of interest are seeded. PCL, in particular, is an FDA-approved biocompatible and bioresorbable scaffold that has been used in the past as a three-dimensional scaffold for craniofacial bone grafts, but is now being used in other applications [171,172]. Our laboratory in particular has used PCL-based scaffolds as model systems in which GFP-labeled triple negative breast cancer cells were cultured [33]. We showed that the cancer cells were capable of infiltrating the depth of the scaffold while proliferating, and were also capable of establishing vascular networks through the formation of blood vessels [33]. Other studies have shown that PCL blended with PLA mimics bone architecture well, whereby osteosarcoma cells were capable of seeding and proliferating in scaffolds generated with these materials [165,173,174]. Thus, biomimetic synthetic polymers are a useful tool that effectively models both the biomechanical properties and architecture of the bone niche. Furthermore, these models allow for the manipulation of the niche via exchange of seeded cells of interest.

##### Bioreactor-Based Engineered Tissue

Bioreactors are another useful tool to specifically examine bone–tumor interactions under long-term culture conditions [118,119,120]. While these models are mainly limited to studying cellular behavior in-vitro, they do offer the flexibility of long-term cell growth/crosstalk (i.e., 6+ months). Vogler, Mastro, and colleagues designed a 3D bioreactor capable of generating crosstalk upon long-term culture (up to 120 days) of both murine and human osteoblast cell lines. Importantly, these cells displayed characteristics of normal osteoblasts throughout their growth [118]. Furthermore, that same model system was used to mimic breast cancer colonization in bone whereby a tri-culture of osteoblasts, osteoclasts, and human breast cancer cells recapitulated events observed during the “vicious cycle of breast cancer metastasis”, including increased osteoclast activity, along with a decrease in osteoblast response, decreased matrix thickness, increased matrix resorption, and increased cancer cell proliferation [119]. Both human and murine cells were capable of growth in the bioreactor.

##### Humanized Biomaterial Implants

Finally, other groups have developed novel humanized biomaterial implants to serve as new platforms for the study of the bone niche. Andreeff and colleagues developed an extramedullary bone (EMB) model using mesenchymal stromal cells and endothelial colony-forming cells to recapitulate the hematopoietic and bone marrow microenvironments [30]. Importantly, this was the first development of a genetically controlled human bone marrow microenvironment capable of engrafting into NOD/SCID/IL-2r gamma null (NSG) immunocompromised mice. Unlike prior models to study the human bone marrow microenvironment, the EMB closely mimics human disease in-vivo, provides a robust hematopoietic environment, and can serve as a representative assay for modeling cancer metastases to bone [30]. Importantly, this model effectively recapitulated leukemia cell seeding and proliferation in a human bone marrow microenvironment, as well as permitted cancer cell metastasis to the ends of long bones; a preferential site of cancer cell invasion [30]. Several years later, Andreeff and colleagues refined this mouse model to include a humanized bone-chip implant whereby freshly isolated human bone fragments were collected from patients undergoing hip replacement surgery and mixed with Matrigel™ [29]. Four weeks post-implantation into the flanks for NSG mice, the human bone implants developed their own vasculature. To investigate leukemia engraftment and the impacts of leukemia on osteogenic differentiation, Molm13 leukemia cells expressing GFP and luciferase were injected via the tail vein and strongly engrafted into the human bone implant as early as 10 days post-injection [29]. Similarly, the Lee group utilized a bioengineering approach to create genetically engineered scaffold microenvironments using human bone stromal cells [27,28]. The human bone marrow stromal cells were genetically engineered to stably express human cytokines (including TNF-alpha) or human growth factors (including VEGF) important for bone remodeling and were embedded into 3D porous hydrogel scaffolds. This permitted the generation of a human soluble factor-enriched microenvironment. The seeded scaffolds were then implanted into immunocompromised mice and stromal cell engraftment and proliferation were investigated [27].

**Figure 1:**
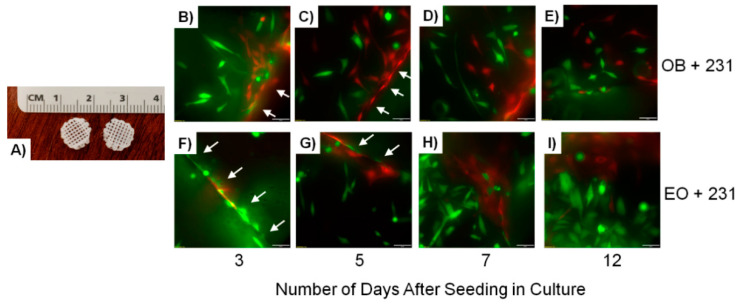
Co-cultures of osteoblasts and breast cancer cells attach and proliferate over time on polycaprolactone printed scaffolds. (**A**) Printed polycaprolactone scaffolds were coated with Matrigel™ then seeded with a 1:1 admix of GFP-MDA-MB-231 human breast cancer cells plus either (**B**–**E**) tdTomato MC3T3-E1 murine osteoblasts (OB) or (**F**–**I**) tdTomato murine educated osteoblast (EO) cells. Seeded scaffolds were imaged for fluorescence over the course of 12 days for cell seeding, growth, and spread on the scaffold. White arrows indicate edges of the criss-cross printed pattern of the scaffold. Scale bar on fluorescent images = 50 μm.

In an effort to merge principles from the EMB model and genetically engineered bone scaffold model, our laboratory seeded PCL scaffolds (Figure 1A) coated with Matrigel™ with GFP-labeled MDA-MB-231 human metastatic breast cancer cells admixed at a 1:1 ratio with either naïve MC3T3-E1 osteoblasts (OB) engineered to express tdTomato or educated osteoblast (EO) cells engineered to express tdTomato [175]. The seeded scaffolds were then cultured for 12 days during which we monitored the engraftment of both the MDA-MB-231GFP breast cancer cells and either MC3T3-E1-tdTomato osteoblasts or tdTomato-EO cells over time using fluorescence imaging. As seen in Figure 1, MDA-MB-231-GFP breast cancer cells and either MC3T3-E1-tdTomato osteoblasts (Figure 1B–E) or tdTomato-EO (Figure 1F–I) cells can clearly be seen proliferating, spreading out, and positioning themselves adjacent to each other within the scaffold over time [175].

**Figure 2:**
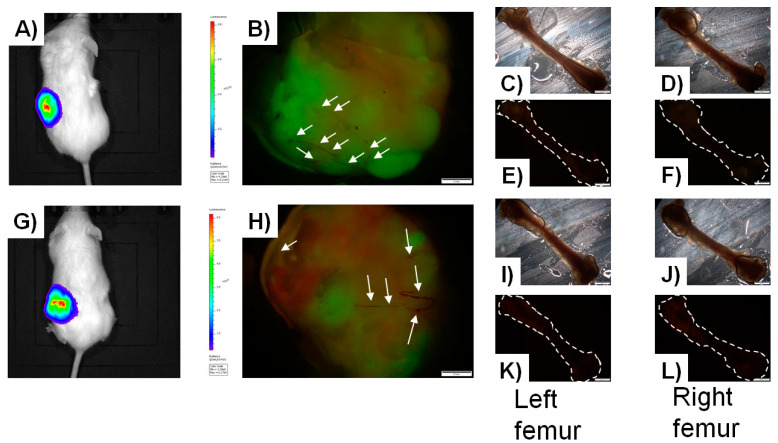
Printed polycaprolactone scaffolds seeded with osteoblasts plus breast cancer cells form tumors in-vivo. IVIS image of a NOD/SCID/IL-2r gamma null (NSG) mouse 2 months post-implant with a printed polycaprolactone scaffold coated with Matrigel™ then seeded with a 1:1 admix of GFP-MDA-MB-231 human breast cancer cells plus either (**A**) tdTomato MC3T3-E1 murine osteoblasts or (**G**) tdTomato murine EO cells. (**B**,**H**) Fluorescent microscope images of tumors from A and G ex-vivo; (**B**) 1:1 admix of GFP-MDA-MB-231 human breast cancer cells plus tdTomato MC3T3-E1 murine osteoblasts; (**H**) 1:1 admix of GFP-MDA-MB-231 human breast cancer cells plus tdTomato murine EO cells. White arrows indicate blood vessel formation in the tumors. Light microscopy images of (**C**,**I**) left and (**D**,**J**) right ex-vivo femurs of (**C**,**D**) NSG mouse implanted with printed polycaprolactone scaffold seeded with 1:1 admix of GFP-MDA-MB-231 human breast cancer cells plus tdTomato MC3T3-E1 murine osteoblasts, or (**I**,**J**) 1:1 admix of GFP-MDA-MB-231 human breast cancer cells plus tdTomato murine EO cells. Fluorescent microscopy images of (**E**,**K**) left and (**F**,**L**) right ex-vivo femurs of (**E**,**F**) NSG mouse implanted with printed polycaprolactone scaffold seeded with 1:1 admix of GFP-MDA-MB-231 human breast cancer cells plus tdTomato MC3T3-E1 murine osteoblasts, or (**K**,**L**) 1:1 admix of GFP-MDA-MB-231 human breast cancer cells plus tdTomato murine EO cells. Scale bar = 2 mm.

Scaffolds were implanted into the flanks of NSG mice 13 days after cell seeding. Mice were monitored via an In-Vivo Imaging System (IVIS) non-invasive intravital imaging for 2 months, where cancer cell proliferation in the scaffold can be seen (Figure 2A,G) [175]. Mice were humanely euthanized, and scaffolds and femurs (site of preferential metastasis) harvested for analysis. Ex-vivo scaffold analysis revealed well-formed tumors composed of either MC3T3-E1-tdTomato osteoblasts (Figure 2B) or tdTomato-EO cells (Figure 2H). Interestingly, even though scaffolds were originally seeded with a 1:1 admix of GFP-cancer cells plus either MC3T3-E1-Tdtomato osteoblasts or tdTomato-EO cells, less proliferation of MC3T3-E1-tdTomato osteoblasts was apparent, as evidenced by less expression of tdTomato: ~38% of the tumor (and ~62% of the tumor composed of GFP-breast cancer cells; Figure 2B), when compared to tdTomato-EO cell engraftment of the tumor: ~55% of the tumor (and ~45% of the tumor composed of GFP-breast cancer cells; Figure 2H) [175]. These results corroborate our prior findings that EO cells suppress metastatic breast cancer cell growth [85]. Both tumors exhibited blood vessel formation (white arrows, Figure 2B,H). Surprisingly however, we were unable to recapitulate breast cancer cell metastasis to the bone, whereby no femurs showed evidence of MDA-MB-231GFP metastatic breast cancer cells by fluorescence microscopy (Figure 2C–F,I–L) [175]. This phenomenon had been observed as part of the EMB model as per Andreeff and colleagues [30].

Whereas these model systems recapitulate select steps of the metastatic cascade or closely model aspects of human disease, none sufficiently combine both a humanized/human bone microenvironment with appropriate stromal cells, architecture, and mechanical forces along with a system that demonstrates all steps of a human metastatic cascade. Thus, three-dimensional bioprinted model systems address this gap in the research.

## 6. Bioprinting

### 6.1. Basis of Bioprinting

Bioprinting provides a novel additive manufacturing technique to fabricate prescribed architectures formed through layer-by-layer construction of biological materials and living cells [176]. Cells can be impregnated into the resultant extracellular matrices post-printing using traditional cell seeding techniques or incorporated directly into the printing process to produce precursor tissue constructs. Such constructs can then be directly implanted in-vivo for regenerative medicine applications or cultured in-vitro to produce mature, biomimetic tissues, holding promise for breast cancer applications [177].

Although bioprinting was initially established in the form of cytoscribing, a modified inkjet printing technique allowing the deposition of biologic materials for cell adhesion and growth into planar geometries that could be individually stacked and adhered [178], the field quickly evolved to produce in-situ three-dimensional structures. Along with developing inkjet techniques, the rise of rapid fabrication processes such as fused deposition modelling (FDM), stereolithography (SLA), and selective laser sintering (SLS) has allowed for more complex three-dimensional shapes [179]. Most recently, the incorporation of living cells into printable biological materials (i.e., bioinks) and modifications in the printing process to maintain cell viability have led to advanced constructs with patterned cellular and structural elements with bioactivity [179]. Thus, current bioprinting technologies allow for an expansive array of promising applications including providing research environments for the study of stem and cancer cell behavior [16,180,181,182], platforms for drug screening, delivery, and production [183,184], and engineered tissues/organs for transplantation [185].

The advantages of bioprinted constructs over traditional two-dimensional cell culture are numerous. The complex, three-dimensional architectures allow for micron-scale precision of the structural environment housing cells, thereby directing their native functions in a manner difficult to achieve with two-dimensional cell culture [186]. Macroscopic geometries can be customized in size and shape identical to native tissues and organs based on patient specific CT and/or MRI data. The biochemical properties of the substrate materials and potential for conjugation with other biomolecules not only support tissue-specific cell function, but can control vascularization, nerve integration, and maturation of engineered tissue [186]. In addition, the recent availability of desktop bioprinting systems with easily accessible hardware and software systems presents a practical option for a fast, low-cost 3D bioactive construct production [187], further supporting the development of personalized tissues for research and regenerative medicine [188].

### 6.2. Bioprinting Techniques

**Figure 3:**
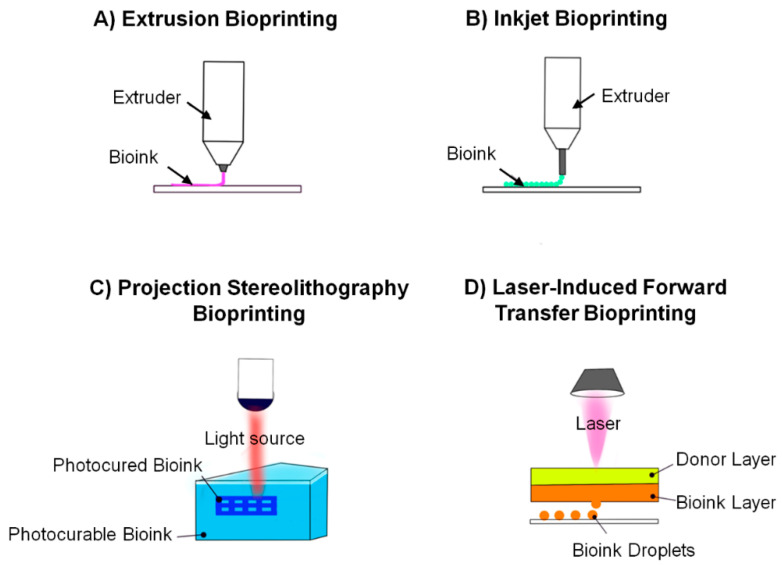
3D bioprinting techniques. (**A**) Extrusion bioprinting: nozzle-based continuous layer-by-layer deposition of bioink using pneumatic pressure-, screw-, or pledged-based mechanisms. (**B**) Inkjet bioprinting: nozzle-based layer-by-layer deposition of bioink droplets using thermal heat or mechanical pulses to acoustic waves. (**C**) Projection stereolithography bioprinting: layer-by-layer photoinduced curing of bioink using UV light. (**D**) Laser-induced forward transfer bioprinting: laser-based depositing bioink droplets through energy transfer from the absorption donor layer to the bioink layer.

3D bioprinting offers the opportunity to manufacture an array of engineered tissues with varying physical and biochemical properties to suit individual applications. Methods for printing include inkjet, extrusion-based laser-induced printing, and projection stereolithography (Figure 3). Each method has unique benefits and limitations and the most appropriate printing technique is dependent on the specific operational requirements of the engineered tissue produced [189].

Extrusion-based manufacturing (Figure 3A) is the most common method of bioprinting, whereby the substrate cell +/− biomaterials are deposited through a thin nozzle in a continuous flowing fashion onto the build plate through the application of force on the bioink reservoir. Various methods for force generation exist, resulting in a range of extrusion-based bioprinter designs, including pneumatic-based (air pressure), piston-driven (vertical force), and screw-driven (rotational force) dispensing techniques [190]. Scaffold constructs can be printed from a homogeneous cell-laden biomaterial ink directly, with dual extrusion heads allowing for independent biomaterial deposition followed by targeted cell placement within the structure, or as a cell-free scaffold with manual cell seeding post-printing [190]. Thus, there is an expanded range of materials that can be used as substrates (e.g., those requiring washing prior to cell seeding to enhance viability) and more precise control over cell distribution with the resultant matrices. The resolution of micro-extrusion bioprinting is in general from 100–500 μm, though it can be as small as 50 μm with the incorporation of microfluidics in the system [179,191]. The extrusion-based method allows for fabrication of structures with bioinks of higher viscosities and cell densities (e.g., greater than 1 × 10^6^ cells/mL [190]), however, as a nozzle-based system it is also associated with shear stress, potentially impacting cell viability [179].

Inkjet bioprinting (Figure 3B), the first method developed, involves dispensing bioink through a thin nozzle in 1–100 μL volumes in a precise three-dimensional pattern [192]. Also known as the drop-on-demand method, the droplets are discharged by either an internal vapor bubble generated by an external thermal heating element or an acoustic wave generated by mechanical pulses of a piezoelectric element allowing for a resolution of 50 μm [177,179]. Printing using this technique is fast (up to 10,000 droplets/second) and relatively low cost vs. other methods, but input substrate options and cell densities may be limited secondary to nozzle clogging with more viscous bioinks [177,179]. Moreover, the heat, acoustic pressure, and high shear rates generated can compromise cell viability, although limiting exposure time may improve cell survival [179,187].

Projection stereolithography (SLA; Figure 3C) is another layer-by-layer fabrication method for bioprinting which involves solidifying liquid biocompatible resins by photopolymerization under irradiation [192,193]. This method is therefore limited to specialized, photocurable bioinks [177]. Although SLA produces mechanically robust constructs, uncured bioink can seep into open spaces, making hollow structures (e.g., vessels) particularly challenging to execute [193].

Laser-induced printing (Figure 3D) does not involve a nozzle, circumventing some of the limitations of the previously described methods. Instead, a glass slide coated with an ink solution and a laser absorption layer consisting of a metal or metal oxide is ablated by a laser at distinct location (40–100 μm resolution), generating a pressure that ejects ink onto the substrate [189]. Also known as laser-induced forward transfer, it can print viscous bioinks at very high resolution, although at very high costs and temperatures that may risk heat-induced cell death [189].

**Figure 4:**
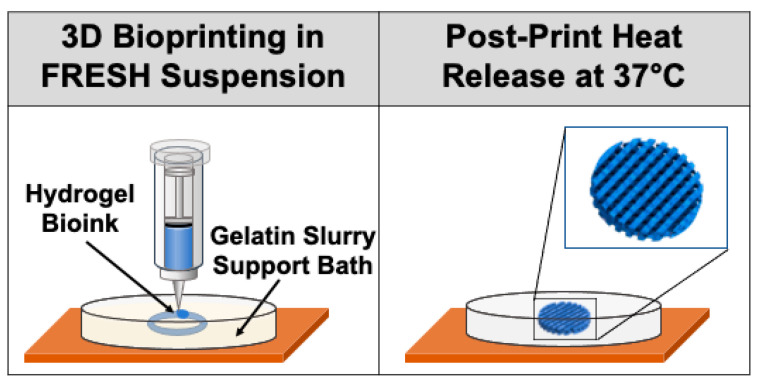
Freeform reversible embedding of suspended hydrogels (FRESH) bioprinting technique. Hydrogel bioink deposited via extrusion bioprinting into a thermoreversible gelatin slurry support bath with a Bingham plastic rheology allows for structural suspension of bioprinted tissue scaffolds to mitigate the force of gravity. An overnight post-print heat release in a 37 °C incubator melts the gelatin, leaving the 3D structure behind.

In an effort to maintain the structural integrity of some complex 3D structures, support material may be necessary to improve the printability of certain bioinks and attain more accurate print results [194]. A suspension-based 3D bioprinting technique, known as freeform reversible embedding of suspended hydrogels (FRESH), allows for the printing of soft hydrogels in a support bath of a thermoreversible gel that can be subsequently melted away, as depicted in Figure 4 [195]. The extrusion and embedding of the bioinks into the gel in essence diminish gravitational effects, allowing for print geometries otherwise unattainable with certain soft biomaterials, as they would ordinarily collapse in air [196]. FRESH-printed structures require some post-print processing, including washing away the excess gelatin post-melting [196]. This noteworthy technique has led to improved print resolution and reliability, and the potential to engineer versatile unsupported print architectures and larger advanced tissue scaffolds [195].

### 6.3. Bioinks

Bioinks are substrates used to produce 3D printed constructs containing cells and/or extracellular matrix components. The composition of the bioink plays a critical role in maintaining cell viability during the printing process and providing the structural integrity necessary to support function post-production [188]. For example, hydrogel-based cell-laden bioinks can protect cells from potentially harmful desiccation and shear forces during the printing [197]. In order to achieve the ideal balance between cell survival and the desired construct structural parameters, careful consideration of not only cytocompatibility, but biocompatibility, bioactivity, and local geometry must be considered to support cell differentiation and growth [189]. Moreover, considerations of printability (e.g., viscosity) along with final overall construct mechanical strength and degradation characteristics (e.g., crosslinking) also require attention when determining the resultant construct physical properties [189].

The three most common bioink options include cell-laden hydrogels, ECM-based inks, and cell suspensions [189]. Cell-laden hydrogels closely mimic/recapitulate the cellular microenvironment by incorporating bioactive compounds and growth factors and permitting the diffusion of nutrients [198]. Natural hydrogels, such as collagen, fibrin, gelatin, and hyaluronic acid are inherently bioactive and simulate the native structure of the ECM, whereas synthetic hydrogels, such as pluronic (poloxamer) and polyethylene glycol (PEG), are mechanically tunable and can support the delivery of added bioactive cues [189,199]. While such hydrogels allow for highly reproducible structures, their limited mechanical strength can be an issue, often augmented by FRESH techniques during and crosslinking after printing the polymers [199].

Single-component hydrogels prepared as bioinks may not adequately provide the composition and function of native ECM in 3D models, as they lack the complex environment that allows for cell engraftment, migration, signaling, and function [200,201]. Decellularized ECM (dECM) offers organ-specific biochemical cues from native ECM to improve cell proliferation and survival [200]. The use of dECM in bioinks can be enhanced by the addition of chemical and biological crosslinking agents to strengthen the scaffold mechanically and improve bioactivity [200]. Maintaining the viscoelastic materials of the tissue is vital to generating a sufficient model of the native tissue. Collagen is a common bioink and major ECM protein as it is easily crosslinked using methods, such as temperature and pH, however, its gelation time and unstable mechanical properties are barriers for use in 3D bioprinting [202]. dECM hydrogels are a promising method for constructing functional tissues and organs with multicellular compositions.

The incorporation of specific bioactive additives allows for manipulation of the mechanical and physiological properties of printed constructs [189]. Hydroxyapatite (HA) and bone morphogenetic protein 2 (BMP-2), two common bioink additives, are known for promoting osteoinductivity and osteoconductivity, and can also confer structural and mechanical properties to bioprinted bone constructs [179]. BMP-2 has been shown to induce mesenchymal stem cells toward osteogenic differentiation [203], and when loaded into hydrogel-based 3D printed constructs, demonstrated sustained release and improved osteogenic differentiation and bone formation effects in-vitro and in-vivo [204].

HA has been shown to regulate the behavior of cells, specifically osteoblast adhesion on nanocrystalline hydroxyapatite (nHA) nanoparticles [205], and to improve normal bone formation, through the stimulation of cell proliferation and osteogenic differentiation [206]. The incorporation of nHA with gelatin methacrylate (GelMA) into bioprinted bone matrices in a study by Xuan Zhou and colleagues showed osteoblast and mesenchymal stem cell (MSC) proliferation and improved overall compressive stress of the resultant matrix constructs [207]. Hyperelastic bone (HB), a recently commercialized extrudable bioink made up of hydroxyapatite and PCL or poly(lactic-co-glycolic acid) offers a promising material for bioprinted bone applications [208]. In addition to its local effect on cell function, printed HB constructs can be successfully stretched, cut, and sutured to a soft tissue (such as tendon), making it a promising candidate for applications in tissue replacement surgeries [208]. Synthetic scaffolds constructed from this porous substrate have been used to study bone regeneration in-vivo [209]. Fluffy–poly(lactic-co-glycolic acid) and hyperelastic bone scaffolds were compared against a negative control and positive control of autologous calvarial bone for the treatment of calvarial defects in rats. The hyperelastic bone was shown to be effective for bone regeneration and for inducing bone formation in-vivo in the defects. Hyperelastic bone has also been implanted in non-human primates to model biocompatibility and fusion rates, and successfully demonstrated vascularization and no significant immune response [210,211].

### 6.4. Applications of Bioprinting in Cancer

#### 6.4.1. 3D Printed In-Vitro Models

Three-dimensional bioprinting presents an opportunity to develop functioning tissue beyond just structural scaffolds by incorporating cells into the printable bioinks [179]. Current applications of bioprinting include three-dimensional extracellular matrix components, organ structures, regenerative tissue grafts, and disease models [198]. FRESH 3D bioprinting can help to optimize the fabricated microenvironments, by controlling print resolution, improving cell viability, and supporting delicate architectures necessary for certain in-vitro cancer research models. For example, Lewicki et al. used the technique to optimize human neuroblastoma cell-laden hydrogels of low viscosity [212]. Applications of dECM-based bioinks hold particular promise in studying ECM components of the tumor microenvironment (TME) and tumor–ECM interplay [213]. Quickly polymerizable dECM bioinks allow for tunable stiffness in applications such as 3D bioprinted kidney cancer constructs in which 3D bioprinted dECM microtumor models were achieved with refined control over both the multicellular populations and dECM bioink deposition [213]. Notably, bioprinted matrices constructed from various substrates with or without FRESH methods can provide viable in-vitro models for understanding cell behaviors and interactions in cancer. For example, extrusion-based printing of bioinks composed of HeLa cells and gelatin/alginate/fibrinogen hydrogels allowed for the development of a 3D cervical tumor model [214]. Compared with cells in planar 2D cultures, HeLa cells in the 3D environment showed more behavioral similarity to native cancer cells, including higher cell proliferation, matrix metalloproteinase (MMP) protein expression, and chemoresistance [215].

Increasing numbers of bioprinted models are being developed as higher fidelity systems to study key aspects of cancer development and progression in a variety of tissues/organs. Tang et al. used bioprinting techniques to develop a complex in-vitro 3D glioblastoma model to allow for exploration of the role of immune components within the tissue microenvironment [216]. The glioblastoma stem cells, resident CNS cells, and macrophage precursors better resembled the invasive cell types of patient tumor tissue in the bioprinted models as opposed to sphere cultures [216]. Hakobyan et al. used laser-assisted bioprinting to generate 3D pancreatic cell spheroid arrays to replicate the initial stages of pancreatic ductal adenocarcinoma (PDAC) as a high-throughput, reproducible model for the study of pancreatic cancer progression and potential therapeutic approaches [217]. Novel model systems are emerging using 3D bioprinting techniques to study other neurological tumors, as well as liver, breast, and skin cancers, that allow for the in-vitro study of susceptibility and resistance to chemotherapeutics for predictable human response [218].

#### 6.4.2. Bioprinted Models for Breast Cancer Metastasis

Bioprinting offers a biomimetic in-vitro three-dimensional cancer culture system capable of modeling cell colonization, tumor growth, and response to therapies. The accuracy of the architecture, vascularization, and composition of the tumor microenvironments demonstrated by bioprinted models reinforces the progress in this domain beyond conventional tissue-engineered constructs. However, cell viability presents a great challenge in the bioprinting process, as the high-throughput processing can directly impact cell proliferation. Recent advances in the use of bioprinting to model cancer microenvironments include tumor fabrication [51,52], allowing for successful modeling of cancer cell interactions.

One of the most crucial elements of an in-vitro cancer model is the scaffold, which emulates the extracellular matrix of the cancer microenvironment. Bioprinted matrices allow for the fabrication of those scaffolds, permitting continued study of the metastasis of breast cancer in bone. In-vitro models provide limitless opportunity for the study of cancer cell interactions and therapeutic advances. Current applications of bioprinting in the study of breast cancer metastasis in bone include bioprinted bone matrices to investigate cancer cell interactions, cell migration, and drug resistance.

Cell-laden bone matrix scaffolds to study the interaction of breast cancer cells with osteoblasts or mesenchymal stromal cells were developed by Zhou and colleagues [37]. The bone matrices were printed with a stereolithography bioprinter, using a bioink consisting of GelMA hydrogel and nanohydroxyapatite (nHA) (incorporated to simulate native bone tissue), and a printable bioink with a cell suspension consisting of osteoblasts or mesenchymal stromal cells. Breast cancer cells were seeded on the surface of the stromal cell-laden matrices and co-cultured with osteoblasts or mesenchymal stromal cells for 5 days. In the co-culture environment, breast cancer cells showed enhanced proliferation but drastically inhibited the osteoblast/mesenchymal stromal cell growth. These data suggest that nHA facilitates interactions between the bone stromal cells and breast cancer cells [37].

**Figure 5:**
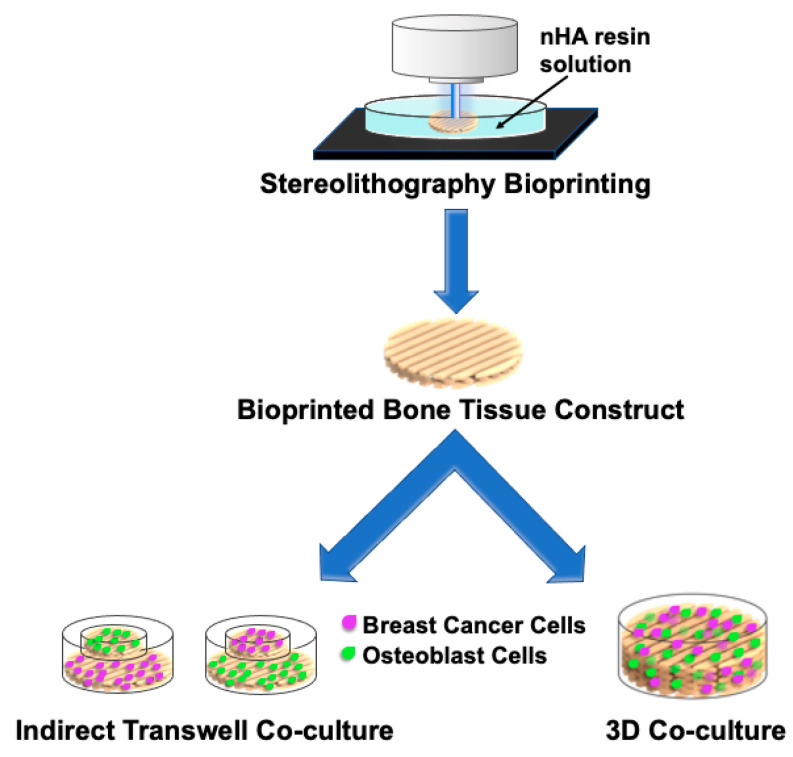
3D bioprinted bone tissue constructs to study crosstalk between osteoblasts and breast cancer cells. Indirect transwell co-culture and 3D co-culture of breast cancer cells and osteoblasts on an artificial nanohydroxyapatite (nHA) bone matrix fabricated by stereolithography-based 3D printing. These model systems permit the study of cellular crosstalk between osteoblasts and breast cancer cells as it relates to cellular morphology, proliferation, osteoblast differentiation, expression of soluble factors, alterations in cellular protein expression, and cellular engraftment into the bone matrix.

Exploring the crosstalk between different cell types, Zhu et al. conducted a study observing the interaction between human fetal osteoblasts (hFOBs) and metastatic breast cancer cells on a 3D bioprinted artificial bone matrix, as depicted in Figure 5 [219]. The matrix also contained the calcium phosphate nHA as it is associated with metastatic breast cancer progression to bone [219]. Human breast cancer cells co-cultured with hFOB cells on the matrix impacted the morphology and proliferation rate of both cell types, in addition to enhanced IL-8 secretion, a pro-inflammatory chemokine that contributes to angiogenesis and tumorigenesis. Notably, the presence of metastatic breast cancer cells induced heightened osteoblastic IL-8 expression. Furthermore, the addition of the nHA to the bone matrix increased the overall proliferation rate of breast cancer cells in a concentration-dependent manner [219]. Beyond examining cellular morphology, proliferation, and cytokine expression, indirect and 3D bioprinted constructs may also be used to assay alterations in cellular crosstalk, including osteoblast differentiation and cellular protein expression, as well as cellular engraftment into the 3D bone matrix (Figure 5). Overall, these data suggest that the composition of the 3D bioprinted matrix is crucial to fully recapitulating cellular in-vivo behavior of metastatic cancer cells in the bone tumor microenvironment.

## 7. Conclusions and Future Exploration

Although 3D bioprinting allows for the precise control of scaffold architecture, these techniques present many challenges in ensuring ideal cell function. Moving forward, physical and biophysical properties of 3D bioprinted scaffolds, including scaffold–matrix stiffness, “tunability” of the scaffold composition, which may include the addition of growth factors and metabolic products native to an endogenous extracellular matrix to facilitate cell growth, porosity of the scaffold to ensure successful cellular attachment and proliferation, alterations in scaffold size, and alterations in scaffold geometry (e.g., honeycomb vs. grid, etc.), as well as other mechanical properties, should be assessed to optimize these factors for successful cellular engraftment and growth [220,221,222,223,224,225]. Furthermore, optimization of a stable scaffold capable of in-vivo implantation with resistance to degradation over time may be critical to the development of long-term viable cellular systems and matrix vascularization.

Interestingly, it has been found that certain scaffold geometries are superior to others with respect to cellular engraftment and viability over time. In particular, Foresti et al. developed a well-defined scaffold with complex architecture that was vascularized and capable of long-term cellular viability [226]. The authors additionally investigated the biological responses of cells seeded on several different scaffolds of complex geometries and determined certain geometries were superior to others for cellular engraftment and proliferation as well as the ability of the scaffold to retain its shape—in some cases for up to 23 months while in culture [226]. Wang et al. additionally investigated how modulating the parameters of a gelatin/alginate hydrogel scaffold, including using various geometries, pore volumes, volume porosity, and surface areas, affected the cell viability, distribution, morphology, proliferation, and expression of cell-specific markers of C3A liver cells [223]. The authors determined that the geometry of PO250+ (fine checkerboard), with a pore size of 250 μm, was optimal towards eliciting strong cell proliferation and viability, as well as causing the expression of the liver-specific mRNA CYP3A4 and protein albumin [223]. With specific reference to the cellular behavior of cancer cells, Hanumantharao et al. carried out an interesting study examining how different topographical features and mechanical properties of PCL scaffolds influenced human ER+ and triple negative breast cancer cells [227]. The authors found that human MCF-7 ER+ breast cancer cells proliferated well on a variety of different scaffold topographies, where the MCF-7 cells had a higher rate of proliferation on scaffolds with low Young’s modulus and stiffness. On the other hand, human MDA-MB-231 triple negative breast cancer cells preferred scaffolds that had a high matrix stiffness [227]. These results suggest that cancer cells respond to changes in scaffold stiffness, mechanical properties, and topography, which should be considered when developing a 3D bioprinted scaffold to model the tumor microenvironment.

Importantly, scaffold “tunability” may be extremely useful when investigating tumor–stromal cell crosstalk and the biophysical properties of the extracellular matrix. Recently, 3D bioprinted scaffolds have been developed that are capable of the controlled release of growth factors that affect angiogenesis and osteogenesis in the bone microenvironment. Specifically, Freeman et al. developed 3D bioprinted constructs to deliver VEGF and BMP-2 with distinct spatiotemporal release profiles to enhance the regeneration of large bone defects [228]. Importantly, the properties of the 3D bioprinted constructs were “tunable” in that (1) the release of VEGF and BMP-2 could be slowed or accelerated, and (2) the distribution of release of VEGF and BMP-2 could be localized or eluted in a spatial gradient [228]. In a similar fashion, Sun et al. developed a 3D bioprinted scaffold loaded with connective tissue growth factor and transforming growth factor beta 3, then seeded with mesenchymal stromal cells and implanted into mice to facilitate the regeneration of an intervertebral disc [229]. Importantly, the authors showed that the reconstructed intervertebral disc exhibited properties similar to a native disc bone, with corresponding histological and immunological phenotypes [229]. Given that 3D bioprinted scaffolds may be loaded with specific growth factors at different concentrations, these models represent a unique and novel way to study interactions between the tumor and stromal cells in different environmental conditions that recapitulate different stages of disease.

Furthermore, 3D bioprinted scaffolds may also be valuable for their ability to both elute drugs into the niche in a sustained manner over time and manipulate the expression of specific factors in the microenvironment. For example, Wu et al. developed a novel biocompatible scaffold for endothelial cell repair in cardiovascular disease [224]. The team loaded dimethyloxalylglycine (DMOG) into biocompatible ink with a final concentration of 30% (*w*/*w*). Over time, in culture with aortic endothelial cells, the scaffold exhibited sustained release of DMOG into the media, which induced the expression of HIF-1 alpha by the aortic endothelial cells. HIF-1 alpha then elicited the transcriptional activation of VEGF, a HIF-1 alpha target gene, suggesting that implantable 3D bioprinted scaffolds loaded with drugs can aid in the repair of endothelial cells in cardiovascular disease [224]. Using the same principle and taking it a step further, the same idea can be applied to manipulating the tumor microenvironment to elicit the expression of certain tumor suppressor molecules, promote an antitumor immune response, or deliver localized therapeutic treatment. The ability to tailor and customize both the environmental response as well as drug loaded into the printed scaffold would be of great benefit to more efficiently target specific cells in the tumor niche over time.

The use of bioprinted constructs introduces a significant high-throughput, low-cost advance in future cancer research that provides a novel perspective for cancer cell growth determinants and potential therapeutics. The incorporation of growth factors and biophysical properties of the ECM could allow researchers to better understand the 3D breast cancer microenvironment through these native-like tumor platforms.

As research progresses towards optimization of the matrix geometry, and more physiologically accurate ECM, researchers come closer to achieving in-vivo conditions for evaluating breast cancer cell behavior and tumor growth. Achieving a matrix that exhibits native characteristics of tumor–stromal cell crosstalk in-vivo will lead to opportunities to study interactions between breast cancer cells and bone stromal cells, metastatic progression, and response to drug therapy. Drug screening and therapeutic cancer drug response testing on 3D bioprinted bone matrices could also reduce the time to screen candidate therapeutics when compared to in-vivo systems and reduce the number of animal models used in testing, thus providing a viable alternative to traditional animal models.

## Data Availability

The data presented in this study are available in Figure 1 and Figure 2.

## References

[1] Lu P., Takai K., Weaver V.M., Werb Z. (2011). Extracellular matrix degradation and remodeling in development and disease. Cold Spring Harb. Perspect. Biol..

[2] Miller R.T. (2017). Mechanical properties of basement membrane in health and disease. Matrix Biol..

[3] Alford A.I., Kozloff K.M., Hankenson K.D. (2015). Extracellular matrix networks in bone remodeling. Int. J. Biochem. Cell Biol..

[4] Gentili C., Cancedda R. (2009). Cartilage and bone extracellular matrix. Curr. Pharm. Des..

[5] (2018). Bone Metastasis: Symptoms and Diagnosis. Metastatic Breast Cancer Symptoms and Diagnosis. https://www.breastcancer.org/symptoms/types/recur_metast/metastic/bone.

[6] Miller K.D., Siegel R.L., Khan R., Jemal A. (2018). Cancer statistics. Cancer Rehabil..

[7] Bussard K.M., Venzon D.J., Mastro A.M. (2010). Osteoblasts are a major source of inflammatory cytokines in the tumor microenvironment of bone metastatic breast cancer. J. Cell. Biochem..

[8] Mastro A.M., Gay C.V., Welch D. (2003). The skeleton as a unique environment for breast cancer cells. Clin. Exp. Metastasis.

[9] Mundy G.R. (2002). Metastasis to bone: Causes, consequences and therapeutic opportunities. Nat. Rev. Cancer.

[10] Phadke P.A., Mercer R.R., Harms J.F., Jia Y., Frost A.R., Jewell J.L., Bussard K.M., Nelson S., Moore C., Kappes J.C. (2006). Kinetics of metastatic breast cancer cell trafficking in bone. Clin. Cancer Res..

[11] Sun Y.-X., Schneider A., Jung Y., Wang J., Dai J., Wang J., Cook K., Osman N.I., Koh-Paige A.J., Shim H. (2004). Skeletal localization and neutralization of the SDF-1(CXCL12)/CXCR4 axis blocks prostate cancer metastasis and growth in osseous sites in vivo. J. Bone Miner. Res..

[12] Lu P., Weaver V.M., Werb Z. (2012). The extracellular matrix: A dynamic niche in cancer progression. J. Cell Biol..

[13] Walker C., Mojares E., Hernández A.D.R. (2018). Role of extracellular matrix in development and cancer progression. Int. J. Mol. Sci..

[14] Eble J.A., Niland S. (2019). The extracellular matrix in tumor progression and metastasis. Clin. Exp. Metastasis.

[15] Leonard F., Godin B. (2016). 3D in vitro model for breast cancer research using magnetic levitation and bioprinting method. Toxic. Assess..

[16] Reid J.A., Mollica P.A., Bruno R.D., Sachs P.C. (2018). Consistent and reproducible cultures of large-scale 3D mammary epithelial structures using an accessible bioprinting platform. Breast Cancer Res..

[17] Wergedal J.E., Mohan S., Lundy M., Baylink D.J. (1990). Skeletal growth factor and other growth factors known to be present in bone matrix stimulate proliferation and protein synthesis in human bone cells. J. Bone Miner. Res..

[18] Morgan M.P., Cooke M.M., Christopherson P.A., Westfall P.R., McCarthy G.M. (2001). Calcium hydroxyapatite promotes mitogenesis and matrix metalloproteinase expression in human breast cancer cell lines. Mol. Carcinog..

[19] Petersen O.W., Rønnov-Jessen L., Howlett A.R., Bissell M.J. (1992). Interaction with basement membrane serves to rapidly distinguish growth and differentiation pattern of normal and malignant human breast epithelial cells. Proc. Natl. Acad. Sci. USA.

[20] Dhurjati R., Krishnan V., Shuman L.A., Mastro A.M., Vogler E.A. (2008). Metastatic breast cancer cells colonize and degrade three-dimensional osteoblastic tissue in vitro. Clin. Exp. Metastasis.

[21] Dhurjati R., Liu X., Gay C.V., Mastro A.M., Vogler E.A. (2006). Extended-Term culture of bone cells in a compartmentalized bioreactor. Tissue Eng..

[22] Krishnan V., Dhurjati R., Vogler E.A., Mastro A.M. (2010). Osteogenesis in vitro: From pre-osteoblasts to osteocytes: A contribution from the Osteobiology Research Group, The Pennsylvania State University. In Vitro Cell. Dev. Biol. Anim..

[23] Krishnan V., Shuman L.A., Sosnoski D.M., Dhurjati R., Vogler E.A., Mastro A.M. (2011). Dynamic interaction between breast cancer cells and osteoblastic tissue: Comparison of Two- and Three-dimensional cultures. J. Cell. Physiol..

[24] Krishnan V., Vogler E.A., Mastro A.M. (2015). Three-Dimensional in vitro model to study osteobiology and osteopathology. J. Cell. Biochem..

[25] Krishnan V., Vogler E.A., Sosnoski D.M., Mastro A.M. (2013). In vitro mimics of bone remodeling and the vicious cycle of cancer in bone. J. Cell. Physiol..

[26] Sosnoski D.M., Krishnan V., Kraemer W.J., Dunn-Lewis C., Mastro A.M. (2012). Changes in cytokines of the bone microenvironment during breast cancer metastasis. Int. J. Breast Cancer.

[27] Lee J., Heckl D., Parekkadan B. (2016). Multiple genetically engineered humanized microenvironments in a single mouse. Biomater. Res..

[28] Lee J., Li M., Milwid J., Dunham J., Vinegoni C., Gorbatov R., Iwamoto Y., Wang F., Shen K., Hatfield K. (2012). Implantable microenvironments to attract hematopoietic stem/cancer cells. Proc. Natl. Acad. Sci. USA.

[29] Battula V.L., Le P.M., Sun J.C., Nguyen K., Yuan B., Zhou X., Sonnylal S., McQueen T., Ruvolo V., Michel K.A. (2017). AML-induced osteogenic differentiation in mesenchymal stromal cells supports leukemia growth. JCI Insight.

[30] Chen Y., Jacamo R., Shi Y.-X., Wang R.-Y., Battula V.L., Konoplev S., Strunk D., Hofmann N.A., Reinisch A., Konopleva M. (2012). Human extramedullary bone marrow in mice: A novel in vivo model of genetically controlled hematopoietic microenvironment. Blood.

[31] Marturano-Kruik A., Nava M.M., Yeager K., Chramiec A., Hao L., Robinson S., Guo E., Raimondi M.T., Vunjak-Novakovic G. (2018). Human bone perivascular niche-on-a-chip for studying metastatic colonization. Proc. Natl. Acad. Sci. USA.

[32] Hao S., Ha L., Cheng G., Wan Y., Xia Y., Sosnoski D.M., Mastro A.M., Zheng S.-Y. (2018). A spontaneous 3D bone-on-a-chip for bone metastasis study of breast cancer cells. Small.

[33] Shupp A.B., Kolb A.D., Bussard K.M., Birbrair A. (2020). Novel Techniques to Study the Bone-Tumor Microenvironment, in Tumor Microenvironment: Advances in Experimental Medicine and Biology.

[34] Belgodere J.A., King C.T., Bursavich J.B., Burow M.E., Martin E.C., Jung J.P. (2018). Engineering breast cancer microenvironments and 3D bioprinting. Front. Bioeng. Biotechnol..

[35] Marks S.C., Odgren P.R., Bilezikian J.P., Raisz L.G., Rodan G.A. (2002). Structure and Development of the Skeleton, in Principles of Bone Biology.

[36] Minguell J.J., Erices A., Conget P. (2001). Mesenchymal stem cells. Exp. Biol. Med..

[37] Augat P., Schorlemmer S. (2006). The role of cortical bone and its microstructure in bone strength. Age Ageing.

[38] Florencio-Silva R., Sasso G.R.d.S., Sasso-Cerri E., Simões M.J., Cerri P.S. (2015). Biology of bone tissue: Structure, function, and factors that influence bone cells. BioMed Res. Int..

[39] Capulli M., Paone R., Rucci N. (2014). Osteoblast and osteocyte: Games without frontiers. Arch. Biochem. Biophys..

[40] Mullender M.M., Van Der Meer D., Huiskes H.R., Lips P. (1996). Osteocyte density changes in aging and osteoporosis. Bone.

[41] Takahashi N., Udagawa N., Takami M., Suda T., Bilezikian J.P., Raisz L.G., Rodan G.A. (2002). Cells of bone: Osteoclast generation. Principles of Bone Biology.

[42] Kanis J.A., McCloskey E.V. (1997). Bone turnover and biochemical markers in malignancy. Cancer.

[43] Wilson S.R., Peters C., Saftig P., Brömme D., Cathepsin K. (2009). Activity-Dependent regulation of osteoclast actin ring formation and bone resorption. J. Biol. Chem..

[44] Blair H.C. (1998). How the osteoclast degrades bone. Bioessays.

[45] Teitelbaum S.L. (2016). Therapeutic implications of suppressing osteoclast formation versus function. Rheumatology.

[46] Stenbeck G. (2002). Formation and function of the ruffled border in osteoclasts. Semin. Cell Dev. Biol..

[47] Hauschka P.V., Mavrakos A.E., Iafrati M.D., Doleman S.E., Klagsbrun M. (1986). Growth factors in bone matrix. Isolation of multiple types by affinity chromatography on heparin-Sepharose. J. Biol. Chem..

[48] Raggatt L.J., Partridge N.C. (2010). Cellular and molecular mechanisms of bone remodeling. J. Biol. Chem..

[49] Dallas S.L., Prideaux M., Bonewald L.F. (2013). The osteocyte: An endocrine cell … and more. Endocr. Rev..

[50] Sottnik J.L., Dai J., Zhang H., Campbell B., Keller E.T. (2015). Tumor-Induced pressure in the bone microenvironment causes osteocytes to promote the growth of prostate cancer bone metastases. Cancer Res..

[51] Mullen C., Haugh M., Schaffler M., Majeska R., McNamara L.M. (2013). Osteocyte differentiation is regulated by extracellular matrix stiffness and intercellular separation. J. Mech. Behav. Biomed. Mater..

[52] Robling A.G., Turner C.H. (2009). Mechanical signaling for bone modeling and remodeling. Crit. Rev. Eukaryot. Gene Expr..

[53] Alberts B., Johnson A., Lewis J., Morgan D., Raff M., Roberts K., Walter P. (2015). Molecular Biology of the Cell.

[54] Wolff J. (1892). Das Gesetz der Transformation der Knochen.

[55] Klein-Nulend J., Bacabac R.G., Bakker A.D. (2012). Mechanical loading and how it affects bone cells: The role of the osteocyte cytoskeleton in maintaining our skeleton. Eur. Cells Mater..

[56] Klein-Nulend J., Van Der Plas A., Semeins C.M., Ajubi N.E., Erangos J.A., Nijweide P.J., Burger E. (1995). Sensitivity of osteocytes to biomechanical stress in vitro. FASEB J..

[57] Mastro A.M., Gay C.V., Welch D., Donahue H.J., Jewell J., Mercer R., DiGirolamo D., Chislock E.M., Guttridge K. (2004). Breast cancer cells induce osteoblast apoptosis: A possible contributor to bone degradation. J. Cell. Biochem..

[58] Josse J., Velard F., Gangloff S. (2015). Staphylococcus aureus vs. osteoblast: Relationship and consequences in osteomyelitis. Front. Cell. Infect. Microbiol..

[59] Sanchez C., DeBerg M.A., Bellahcène A., Castronovo V., Msika P., Delcour J.P., Crielaard J.M., Henrotin Y. (2008). Phenotypic characterization of osteoblasts from the sclerotic zones of osteoarthritic subchondral bone. Arthritis Rheum..

[60] Sanchez C., Mazzucchelli G., Lambert C., Comblain F., Depauw E., Henrotin Y. (2018). Comparison of secretome from osteoblasts derived from sclerotic versus non-sclerotic subchondral bone in OA: A pilot study. PLoS ONE.

[61] Yoneda T. (1996). Mechanisms of preferential metastasis of breast cancer to bone—(Review). Int. J. Oncol..

[62] Lentino J.R. (2003). Prosthetic joint infections: Bane of orthopedists, challenge for infectious disease specialists. Clin. Infect. Dis..

[63] Berendt T., Byren I. (2004). Bone and joint infection. Clin. Med..

[64] Tillander J., Hagberg K., Berlin Ö., Hagberg L., Brånemark R. (2017). Osteomyelitis risk in patients with transfemoral amputations treated with osseointegration prostheses. Clin. Orthop. Relat. Res..

[65] Kellesarian S.V., Javed F., Romanos G.E. (2018). Osteomyelitis arising around osseointegrated dental implants: A systematic review. Implant Dent..

[66] Semel G., Wolff A., Shilo D., Akrish S., Emodi O., Rachmiel A. (2016). Mandibular osteomyelitis associated with dental implants. A case series. Eur. J. Oral Implantol..

[67] Bosse M.J., Gruber H.E., Ramp W.K. (2005). Internalization of bacteria by osteoblasts in a patient with recurrent, long-term osteomyelitis: A case report. JBJS Case Connect..

[68] Dapunt U., Maurer S., Giese T., Gaida M.M., Hänsch G.M. (2014). The macrophage inflammatory proteins MIP1α(CCL3) and MIP2α(CXCL2) in implant-associated osteomyelitis: Linking inflammation to bone degradation. Mediat. Inflamm..

[69] Bost K.L., Bento J.L., Ellington J.K., Marriott I., Hudson M.C. (2000). Induction of colony-stimulating factor expression following staphylococcus or salmonellainteraction with mouse or human osteoblasts. Infect. Immun..

[70] Wright K.M., Friedland J.S. (2004). Regulation of chemokine gene expression and secretion in Staphylococcus aureus-infected osteoblasts. Microbes Infect..

[71] Ning R., Zhang X., Guo X., Li Q. (2011). Staphylococcus aureus regulates secretion of interleukin-6 and monocyte chemoattractant protein-1 through activation of nuclear factor kappaB signaling pathway in human osteoblasts. Braz. J. Infect. Dis..

[72] Gasper N.A., Petty C.C., Schrum L.W., Marriott I., Bost K.L. (2002). Bacterium-Induced CXCL10 secretion by osteoblasts can be mediated in part through toll-like receptor 4. Infect. Immun..

[73] Somayaji S.N., Ritchie S., Sahraei M., Marriott I., Hudson M.C. (2008). Staphylococcus aureus induces expression of receptor activator of NF-kappaB ligand and prostaglandin E2 in infected murine osteoblasts. Infect. Immun..

[74] Widaa A., Claro T., Foster T.J., O’Brien F.J., Kerrigan S.W. (2012). Staphylococcus aureus protein a plays a critical role in mediating bone destruction and bone loss in osteomyelitis. PLoS ONE.

[75] Sanchez C., Pesesse L., Gabay O., Delcour J.-P., Msika P., Baudouin C., Henrotin Y. (2012). Regulation of subchondral bone osteoblast metabolism by cyclic compression. Arthritis Rheum..

[76] Bianco D., Todorov A., Cengic T., Pagenstert G., Schären S., Netzer C., Hügle T., Geurts J. (2018). Alterations of subchondral bone progenitor cells in human knee and hip osteoarthritis lead to a bone sclerosis phenotype. Int. J. Mol. Sci..

[77] Martineau X., Abed É., Martel-Pelletier J., Pelletier J.-P., Lajeunesse D. (2017). Alteration of Wnt5a expression and of the non-canonical Wnt/PCP and Wnt/PKC-Ca2+ pathways in human osteoarthritis osteoblasts. PLoS ONE.

[78] Kinder M., Chislock E.M., Bussard K.M., Shuman L., Mastro A.M. (2008). Metastatic breast cancer induces an osteoblast inflammatory response. Exp. Cell Res..

[79] Bussard K.M., Gay C.V., Mastro A.M. (2007). The bone microenvironment in metastasis; what is special about bone?. Cancer Metastasis Rev..

[80] Logothetis C., Morris M.J., Den R., Coleman R.E. (2018). Current perspectives on bone metastases in castrate-resistant prostate cancer. Cancer Metastasis Rev..

[81] Logothetis C.J., Lin S.-H. (2005). Osteoblasts in prostate cancer metastasis to bone. Nat. Rev. Cancer.

[82] Roodman G.D. (2004). Mechanisms of bone metastasis. N. Engl. J. Med..

[83] Paget S. (1889). The distribution of secondary growths in cancer of the breast. Lancet.

[84] Paget S. (1989). The distribution of secondary growths in cancer of the breast. Cancer Metastasis Rev..

[85] Kolb A.D., Shupp A.B., Mukhopadhyay D., Marini F.C., Bussard K.M. (2019). Osteoblasts are “educated” by crosstalk with metastatic breast cancer cells in the bone tumor microenvironment. Breast Cancer Res..

[86] Lawson M.A., McDonald M.M., Kovacic N., Khoo W.H., Terry R.L., Down J.M., Kaplan W., Paton-Hough J., Fellows C., Pettitt J.A. (2015). Osteoclasts control reactivation of dormant myeloma cells by remodelling the endosteal niche. Nat. Commun..

[87] Mundy G.R. (1999). Bone Remodeling and Its Disorders.

[88] Kennecke H., Yerushalmi R., Woods R., Cheang M.C.U., Voduc D., Speers C.H., Nielsen T.O., Gelmon K. (2010). Metastatic behavior of breast cancer subtypes. J. Clin. Oncol..

[89] Liede A., Jerzak K.J., Hernandez R.K., Wade S.W., Sun P., Narod S.A. (2016). The incidence of bone metastasis after early-stage breast cancer in Canada. Breast Cancer Res. Treat..

[90] Siegel R.L., Miller K.D.M., Jemal A. (2018). Cancer statistics, 2018. CA Cancer J. Clin..

[91] Lipton A., Uzzo R., Amato R.J., Ellis G.K., Hakimian B., Roodman G.D., Smith M.R. (2009). The science and practice of bone health in oncology: Managing bone loss and metastasis in patients with solid tumors. J. Natl. Compr. Cancer Netw..

[92] Manders K., Van De Poll-Franse L.V., Creemers G.-J., Vreugdenhil G., Van Der Sangen M.J.C., Nieuwenhuijzen G.A.P., Roumen R.M.H., Voogd A.C. (2006). Clinical management of women with metastatic breast cancer: A descriptive study according to age group. BMC Cancer.

[93] Coleman R.E. (1997). Skeletal complications of malignancy. Cancer.

[94] Guise T.A., Mundy G.R. (1998). Cancer and bone. Endocr. Rev..

[95] Guise T.A., Mohammad K.S., Clines G., Stebbins E.G., Wong D.H., Higgins L.S., Vessella R., Corey E., Padalecki S., Suva L. (2006). Basic mechanisms responsible for osteolytic and osteoblastic bone metastases: Fig. 1. Clin. Cancer Res..

[96] Guise T.A. (2000). Molecular mechanisms of osteolytic bone metastases. Cancer.

[97] Marathe D.D., Marathe A., Mager D.E. (2011). Integrated model for denosumab and ibandronate pharmacodynamics in postmenopausal women. Biopharm. Drug Dispos..

[98] Anagnostis P., Vakalopoulou S., Christoulas D., Paschou S.A., Papatheodorou A., Garipidou V., Kokkoris P., Terpos E. (2017). The role of sclerostin/dickkopf-1 and receptor activator of nuclear factor kB ligand/osteoprotegerin signalling pathways in the development of osteoporosis in patients with haemophilia A and B: A cross-sectional study. Haemophilia.

[99] Geng C.-J., Liang Q., Zhong J.-H., Zhu M., Meng F.-Y., Wu N., Liang R., Yuan B.-Y. (2015). Ibandronate to treat skeletal-related events and bone pain in metastatic bone disease or multiple myeloma: A meta-analysis of randomised clinical trials. BMJ Open.

[100] Tu K.N., Lie J.D., Wan C.K.V., Cameron M., Austel A.G., Nguyen J.K., Van K., Hyun D. (2018). Osteoporosis: A review of treatment options. Pharm. Ther..

[101] Greenberg A.J., Rajkumar S.V., Therneau T.M., Singh P., Dispenzieri A., Kumar S.K. (2014). Relationship between initial clinical presentation and the molecular cytogenetic classification of myeloma. Leukemia.

[102] Marino S., Roodman G.D. (2018). Multiple myeloma and bone: The fatal interaction. Cold Spring Harb. Perspect. Med..

[103] Bataille R., Chappard D., Marcelli C., Dessauw P., Sany J., Baldet P., Alexandre C. (1989). Mechanisms of bone destruction in multiple myeloma: The importance of an unbalanced process in determining the severity of lytic bone disease. J. Clin. Oncol..

[104] Ehrlich L.A., Chung H.Y., Ghobrial I., Choi S.J., Morandi F., Colla S., Rizzoli V., Roodman G.D., Giuliani N. (2005). IL-3 is a potential inhibitor of osteoblast differentiation in multiple myeloma. Blood.

[105] Silbermann R., Bolzoni M., Storti P., Guasco D., Bonomini S., Zhou D., Wu J., Anderson J.L., Windle J.J., Aversa F. (2014). Bone marrow monocyte-/macrophage-derived activin A mediates the osteoclastogenic effect of IL-3 in multiple myeloma. Leukemia.

[106] Delgado-Calle J., Anderson J., Cregor M.D., Condon K.W., Kuhstoss S.A., Plotkin L.I., Bellido T., Roodman G.D. (2017). Genetic deletion of Sost or pharmacological inhibition of sclerostin prevent multiple myeloma-induced bone disease without affecting tumor growth. Leukemia.

[107] Waning D.L., Mohammad S.K., Reiken S., Xie W., Andersson D.C., John S., Chiechi A., Wright L.E., Umanskaya A., Niewolna M. (2015). Excess TGF-beta mediates muscle weakness associated with bone metastases in mice. Nat. Med..

[108] Nyman J.S., Merkel A.R., Uppuganti S., Nayak B., Rowland B., Makowski A.J., Oyajobi B.O., Sterling J.A. (2016). Combined treatment with a transforming growth factor beta inhibitor (1D11) and bortezomib improves bone architecture in a mouse model of myeloma-induced bone disease. Bone.

[109] D’Souza S., Del Prete D., Jin S., Sun Q., Huston A.J., Kostov F.E., Sammut B., Hong C.-S., Anderson J.L., Patrene K.D. (2011). Gfi1 expressed in bone marrow stromal cells is a novel osteoblast suppressor in patients with multiple myeloma bone disease. Blood.

[110] Pozzi S., Fulciniti M., Yan H., Vallet S., Eda H., Patel K., Santo L., Cirstea D., Hideshima T., Schirtzinge L. (2013). In vivo and in vitro effects of a novel anti-Dkk1 neutralizing antibody in multiple myeloma. Bone.

[111] Fulciniti M., Tassone P., Hideshima T., Vallet S., Nanjappa P., Ettenberg S.A., Shen Z., Patel N., Tai Y.-T., Chauhan D. (2009). Anti-DKK1 mAb (BHQ880) as a potential therapeutic agent for multiple myeloma. Blood.

[112] Morris M.J., Scher H.I. (2003). Clinical approaches to osseous metastases in prostate cancer. Oncologist.

[113] Wang N., Docherty F.E., Brown H.K., Reeves K.J., Fowles A.C.M., Ottewell P.D., Dear T.N., Holen I., Croucher P.I., Eaton C.L. (2014). Prostate cancer cells preferentially home to osteoblast-rich areas in the early stages of bone metastasis: Evidence from in vivo models. J. Bone Miner. Res..

[114] Roudier M.P., Morrissey C., True L.D., Higano C.S., Vessella R.L., Ott S.M. (2008). Histopathological assessment of prostate cancer bone osteoblastic metastases. J. Urol..

[115] Eastham J.A. (2007). Bone health in men receiving androgen deprivation therapy for prostate cancer. J. Urol..

[116] Sekita A., Matsugaki A., Nakano T. (2017). Disruption of collagen/apatite alignment impairs bone mechanical function in osteoblastic metastasis induced by prostate cancer. Bone.

[117] Matsugaki A., Aramoto G., Ninomiya T., Sawada H., Hata S., Nakano T. (2015). Abnormal arrangement of a collagen/apatite extracellular matrix orthogonal to osteoblast alignment is constructed by a nanoscale periodic surface structure. Biomaterials.

[118] Charhon S.A., Chapuy M.C., Delvin E.E., Valentin-Opran A., Edouard C.M., Meunier P.J. (1983). Histomorphometric analysis of sclerotic bone metastases from prostatic carcinoma with special reference to osteomalacia. Cancer.

[119] Clarke N.W., McClure J., George N.J.R. (1991). Morphometric evidence for bone resorption and replacement in prostate cancer. BJU Int..

[120] Wan X., Corn P.G., Yang J., Palanisamy N., Starbuck M.W., Efstathiou E., Li-Ning-Tapia E.M., Zurita A.J., Aparicio A., Ravoori M.K. (2014). Prostate cancer cell–stromal cell crosstalk via FGFR1 mediates antitumor activity of dovitinib in bone metastases. Sci. Transl. Med..

[121] Fizazi K., Yang J., Peleg S., Sikes C.R., Kreimann E.L., Daliani D., Olive M., Raymond K.A., Janus T.J., Logothetis C. (2003). Prostate cancer cells-osteoblast interaction shifts expression of growth/survival-related genes in prostate cancer and reduces expression of osteoprotegerin in osteoblasts. Clin. Cancer Res..

[122] Oberneder R., Riesenberg R., Kriegmair M., Bitzer U., Klammert R., Schneede P., Hofstetter A., Pantel K. (1994). Immunocytochemical detection and phenotypic characterization of micrometastatic tumour cells in bone marrow of patients with prostate cancer. Urol. Res..

[123] Ottewell P.D. (2016). The role of osteoblasts in bone metastasis. J. Bone Oncol..

[124] Carducci M.A., Nelson J.B., Bowling M.K., Rogers T., Eisenberger M.A., Sinibaldi V., Donehower R., Leahy T.L., Carr R.A., Isaacson J.D. (2002). Atrasentan, an endothelin-receptor antagonist for refractory adenocarcinomas: Safety and pharmacokinetics. J. Clin. Oncol..

[125] Carducci M.A., Padley R.J., Breul J., Vogelzang N.J., Zonnenberg B.A., Daliani D.D., Schulman C.C., Nabulsi A.A., Humerickhouse R.A., Weinberg M.A. (2003). Effect of endothelin-a receptor blockade with atrasentan on tumor progression in men with hormone-refractory prostate cancer: A randomized, phase ii, placebo-controlled trial. J. Clin. Oncol..

[126] Suominen M.I., Fagerlund K.M., Rissanen J.P., Konkol Y.M., Morko J.P., Peng Z., Alhoniemi E.J., Laine S.K., Corey E., Mumberg D. (2017). Radium-223 inhibits osseous prostate cancer growth by dual targeting of cancer cells and bone microenvironment in mouse models. Clin. Cancer Res..

[127] Popper H.H. (2016). Progression and metastasis of lung cancer. Cancer Metastasis Rev..

[128] Baker J., Falconer A.M.D., Wilkinson D.J., Europe-Finner G.N., Litherland G.J., Rowan A.D. (2018). Protein kinase D3 modulates MMP1 and MMP13 expression in human chondrocytes. PLoS ONE.

[129] Tang C.-H., Tan T.-W., Fu W.-M., Yang R.-S. (2007). Involvement of matrix metalloproteinase-9 in stromal cell-derived factor-1/CXCR4 pathway of lung cancer metastasis. Carcinogenesis.

[130] Sugiura H., Yamada K., Sugiura T., Hida T., Mitsudomi T. (2008). Predictors of surivival in patients with bone metastasis of lung cancer. Clin. Ortho. Relat. Res..

[131] Coleman R. (2001). Metastatic bone disease: Clinical features, pathophysiology and treatment strategies. Cancer Treat. Rev..

[132] Hill C.A. (1984). Bronchioloalveolar carcinoma: A review. Radiology.

[133] Gordon J.A.R., Tye C.E., Sampaio A.V., Underhill T.M., Hunter G.K., Goldberg H.A. (2007). Bone sialoprotein expression enhances osteoblast differentiation and matrix mineralization in vitro. Bone.

[134] Kang E.J., Lee S.Y., Kim H.J., Min K.H., Hur G.-Y., Shim J.J., Kang K.H., Oh S.C., Seo J.H., Lee S. (2016). Prognostic factors and skeletal-related events in patients with small cell lung cancer with bone metastases at the time of diagnosis. Oncologist.

[135] Lang J., Zhao Q., He Y., Yu X. (2018). Bone turnover markers and novel biomarkers in lung cancer bone metastases. Biomarkers.

[136] Papotti M., Kalebic T., Volante M., Chiusa L., Bacillo E., Cappia S., Lausi P.O., Novello S., Borasio P., Scagliotti G.V. (2006). Bone sialoprotein is predictive of bone metastases in resectable non–small-cell lung cancer: A retrospective case-control study. J. Clin. Oncol..

[137] Brown J.E., Cook R.J., Major P., Lipton A., Saad F., Smith M.R., Lee K.-A., Zheng M., Hei Y.-J., Coleman R.E. (2005). Bone turnover markers as predictors of skeletal complications in prostate cancer, lung cancer, and other solid tumors. J. Natl. Cancer Inst..

[138] Costa L., Demers L.M., Gouveia-Oliveira A., Schaller J., Costa E.B., De Moura M.C., Lipton A. (2002). Prospective evaluation of the peptide-bound collagen type I cross-links N-telopeptide and C-telopeptide in predicting bone metastases status. J. Clin. Oncol..

[139] Liu B., Zhao Y., Yuan J., Zeng L., Sun R., Meng X., Yang S. (2017). Elevated N-telopeptide as a potential diagnostic marker for bone metastasis in lung cancer: A meta-analysis. PLoS ONE.

[140] Zhang Y., Yan S., Su Y., Chen H., Wang S., Sun B., Zhang L. (2018). Serum cross-linked N-telopeptide of type I collagen as a biomarker of bone metastases for patients with lung cancer: A meta-analysis. Int. J. Clin. Exp. Med..

[141] Hu Z., Lin D., Yuan J., Xiao T., Zhang H., Sun W., Han N., Ma Y., Di X., Gao M. (2005). Overexpression of osteopontin is associated with more aggressive phenotypes in human non-small cell lung cancer. Clin. Cancer Res..

[142] Kothari A.N., Arffa M., Chang V., Blackwell R.H., Syn W.-K., Zhang J., Mi Z., Kuo P.C. (2016). Osteopontin—A master regulator of epithelial-mesenchymal transition. J. Clin. Med..

[143] Aguirre-Ghiso J.A. (2007). Models, mechanisms, and clinical evidence for cancer dormancy. Nat. Rev. Cancer.

[144] Braun S., Naume B. (2005). Circulating and disseminated tumor cells. J. Clin. Oncol..

[145] Braun S., Vogl F.D., Naume B., Janni W., Osborne M.P., Coombes R.C., Schlimok G., Diel I.J., Gerber B., Gebauer G. (2005). A pooled analysis of bone marrow micrometastasis in breast cancer. N. Engl. J. Med..

[146] Quayle L., Ottewell P.D., Holen I. (2015). Bone metastasis: Molecular mechanisms implicated in tumour cell dormancy in breast and prostate cancer. Curr. Cancer Drug Targets.

[147] Scimeca M., Antonacci C., Toschi N., Giannini E., Bonfiglio R., Buonomo C.O., Pistolese C.A., Tarantino U., Bonanno E. (2018). Breast osteoblast-like cells: A reliable early marker for bone metastases from breast cancer. Clin. Breast Cancer.

[148] Bodenstine T.M., Beck B.H., Cao X., Cook L.M., Ismail A., Powers S.J.K., Powers J.K., Mastro A.M., Welch D. (2011). Pre-osteoblastic MC3T3-E1 cells promote breast cancer growth in bone in a murine xenograft model. Chin. J. Cancer.

[149] Yumoto K., Eber M.R., Wang J., Cackowski F.C., Decker A.M., Lee E., Nobre A.R., Aguirre-Ghiso J.A., Jung Y., Taichman R.S. (2016). Axl is required for TGF-beta2-induced dormancy of prostate cancer cells in the bone marrow. Sci. Rep..

[150] Kobayashi A., Okuda H., Xing F., Pandey P.R., Watabe M., Hirota S., Pai S.K., Liu W., Fukuda K., Chambers C. (2011). Bone morphogenetic protein 7 in dormancy and metastasis of prostate cancer stem-like cells in bone. J. Exp. Med..

[151] Weigelt B., Ghajar C.M., Bissell M.J. (2014). The need for complex 3D culture models to unravel novel pathways and identify accurate biomarkers in breast cancer. Adv. Drug Deliv. Rev..

[152] Gómez-Cuadrado L., Tracey N., Ma R., Qian B., Brunton V.G. (2017). Mouse models of metastasis: Progress and prospects. Dis. Model. Mech..

[153] Saxena M., Christofori G. (2013). Rebuilding cancer metastasis in the mouse. Mol. Oncol..

[154] Ghajar C.M., Peinado H., Mori H., Matei I.R., Evason K.J., Brazier H., De Almeida D.L., Koller A., Hajjar K.A., Stainier D.Y.R. (2013). The perivascular niche regulates breast tumour dormancy. Nat. Cell Biol..

[155] Naumov G.N., Macdonald I.C., Weinmeister P.M., Kerkvliet N., Nadkarni K.V., Wilson S.M., Morris V.L., Groom A.C., Chambers A.F. (2002). Persistence of solitary mammary carcinoma cells in a secondary site: A possible contributor to dormancy. Cancer Res..

[156] Chen Y.-C., Mastro A.M., Sosnoski D.M., Norgard R.J., Grove C.D., Vogler E.A. (2014). Abstract 4891: Dormancy and growth of metastatic breast cancer cells in a bone-like microenvironment. Tumor Biol..

[157] Angeloni V., Negrini N.C., De Marco C., Bertoldi S., Tanzi M.C., Daidone M.G., Farè S. (2017). Polyurethane foam scaffold as in vitro model for breast cancer bone metastasis. Acta Biomater..

[158] Khanna C., Hunter K. (2005). Modeling metastasis in vivo. Carcinogenesis.

[159] Clarke R.B. (1996). Human breast cancer cell line xenografts as models of breast cancer—The immunobiologies of recipient mice and the characteristics of several tumorigenic cell lines. Breast Cancer Res. Treat..

[160] Wright L.E., Ottewell P.D., Rucci N., Peyruchaud O., Pagnotti G.M., Chiechi A., Buijs J.T., Sterling J.A. (2016). Murine models of breast cancer bone metastasis. BoneKEy Rep..

[161] Shupp A.B., Kolb A.D., Bussard K.M. (2020). Novel techniques to study the bone-tumor microenvironment. Adv. Exp. Med. Biol..

[162] Kang Y., Siegel P.M., Shu W., Drobnjak M., Kakonen S.M., Cordón-Cardo C., Guise T.A., Massagué J. (2003). A multigenic program mediating breast cancer metastasis to bone. Cancer Cell.

[163] Yoneda T., Williams P.J., Hiraga T., Niewolna M., & Nishimura R. (2001). A bone-seeking clone exhibits different biological properties from the MDA-MD-231 parental human breast cancer cells and a brain-seeking clone in vivo and in vitro. J. Bone Miner Res..

[164] Cassereau L., Miroshnikova Y.A., Ou G., Lakins J., Weaver V.M. (2015). A 3D tension bioreactor platform to study the interplay between ECM stiffness and tumor phenotype. J. Biotechnol..

[165] Matei I., Rampersaud S., Lyden D. (2018). Engineered niches model the onset of metastasis. Nat. Biomed. Eng..

[166] Vanderburgh J.P., Guelcher S.A., Sterling J.A. (2018). 3D bone models to study the complex physical and cellular interactions between tumor and the bone microenvironment. J. Cell. Biochem..

[167] Caliari S.R., Burdick J.A. (2016). A practical guide to hydrogels for cell culture. Nat. Methods.

[168] Page J.M., Merkel A.R., Ruppender N.S., Guo R., Dadwal U.C., Cannonier S., Basu S., Guelcher S.A., Sterling J.A. (2015). Matrix rigidity regulates the transition of tumor cells to a bone-destructive phenotype through integrin B3 and TGF-B receptor type II. Biomaterials.

[169] Kimura Y., Matsugaki A., Sekita A., Nakano T. (2017). Alteration of osteoblast arrangement via direct attack by cancer cells: New insights into bone metastasis. Sci. Rep..

[170] Tibbitt M.W., Anseth K.S. (2009). Hydrogels as extracellular matrix mimics for 3D cell culture. Biotechnol. Bioeng..

[171] Hung B.P., Naved B.A., Nyberg E.L., Dias M., Holmes C.A., Elisseeff J.H., Dorafshar A.H., Grayson W. (2016). Three-Dimensional printing of bone extracellular matrix for craniofacial regeneration. ACS Biomater. Sci. Eng..

[172] Temple J.P., Hutton D.L., Hung B.P., Huri P.Y., Cook C.A., Kondragunta R., Jia X., Grayson W. (2014). Engineering anatomically shaped vascularized bone grafts with hASCs and 3D-printed PCL scaffolds. J. Biomed. Mater. Res. Part A.

[173] Carpenter R.A., Kwak J.-G., Peyton S.R., Lee J. (2018). Implantable pre-metastatic niches for the study of the microenvironmental regulation of disseminated human tumour cells. Nat. Biomed. Eng..

[174] Patricio T., Domingos M., Gloria A., Bártolo P. (2013). Characterisation of PCL and PCL/PLA scaffolds for tissue engineering. Procedia CIRP.

[175] Kolb A.D., Shupp A.B., Bussard K.M. (2021). Unpublished Data.

[176] Dababneh A.B., Ozbolat I.T. (2014). Bioprinting technology: A current state-of-the-art review. J. Manuf. Sci. Eng..

[177] Cleversey C., Robinson M., Willerth S.M. (2019). 3D printing breast tissue models: A review of past work and directions for future work. Micromachines.

[178] Klebe R.J. (1988). Cytoscribing: A method for micropositioning cells and the construction of two- and three-dimensional synthetic tissues. Exp. Cell Res..

[179] Ashammakhi N., Hasan A., Kaarela O., Byambaa B., Sheikhi A., Gaharwar A.K., Khademhosseini A. (2019). Advancing frontiers in bone bioprinting. Adv. Healthc. Mater..

[180] Gao G., Hubbell K., Schilling A.F., Dai G., Cui X. (2017). Bioprinting cartilage tissue from mesenchymal stem cells and PEG hydrogel. Toxic. Assess..

[181] Gao G., Schilling A.F., Yonezawa T., Wang J., Dai G., Cui X. (2014). Bioactive nanoparticles stimulate bone tissue formation in bioprinted three-dimensional scaffold and human mesenchymal stem cells. Biotechnol. J..

[182] Ling K., Huang G., Liu J., Zhang X., Ma Y., Lu T., Xu F. (2015). Bioprinting-Based high-throughput fabrication of three-dimensional MCF-7 human breast cancer cellular spheroids. Engineering.

[183] Mirani B., Pagan E., Shojaei S., Duchscherer J., Toyota B.D., Ghavami S., Akbari M. (2019). A 3D bioprinted hydrogel mesh loaded with all-trans retinoic acid for treatment of glioblastoma. Eur. J. Pharmacol..

[184] Wei X., Liu C., Wang Z., Luo Y. (2020). 3D printed core-shell hydrogel fiber scaffolds with NIR-triggered drug release for localized therapy of breast cancer. Int. J. Pharm..

[185] Yu Y., Zhang Y., Ozbolat I.T. (2014). A hybrid bioprinting approach for scale-up tissue fabrication. J. Manuf. Sci. Eng..

[186] Gao G., Cui X. (2015). Three-Dimensional bioprinting in tissue engineering and regenerative medicine. Biotechnol. Lett..

[187] Murphy S.V., Atala A. (2014). 3D bioprinting of tissues and organs. Nat. Biotechnol..

[188] Gudapati H., Dey M., Ozbolat I.T. (2016). A comprehensive review on droplet-based bioprinting: Past, present and future. Biomaterials.

[189] Ji S., Guvendiren M. (2017). Recent advances in bioink design for 3D bioprinting of tissues and organs. Front. Bioeng. Biotechnol..

[190] Placone J.K., Engler A.J. (2017). Recent advances in extrusion-based 3D printing for biomedical applications. Adv. Healthc. Mater..

[191] Colosi C., Shin S.R., Manoharan V., Massa S., Costantini M., Barbetta A., Dokmeci M.R., Dentini M., Khademhosseini A. (2016). Microfluidic bioprinting of heterogeneous 3D tissue constructs using low-viscosity bioink. Adv. Mater..

[192] Ding S., Feng L., Wu J., Zhu F., Yao R., Tan Z. (2018). Bioprinting of stem cells: Interplay of bioprinting process, bioinks, and stem cell properties. ACS Biomater. Sci. Eng..

[193] Skoog S.A., Goering P.L., Narayan R.J. (2014). Stereolithography in tissue engineering. J. Mater. Sci. Mater. Med..

[194] Li J., Wu C., Chu P.K., Gelinsky M. (2020). 3D printing of hydrogels: Rational design strategies and emerging biomedical applications. Mater. Sci. Eng. Rep..

[195] Lee A., Hudson A., Shiwarski D.J., Tashman J.W., Hinton T.J., Yerneni S.S., Bliley J.M., Campbell P.G., Feinberg A.W. (2019). 3D bioprinting of collagen to rebuild components of the human heart. Science.

[196] Mirdamadi E., Tashman J.W., Shiwarski D.J., Palchesko R.N., Feinberg A.W. (2020). Fresh 3D bioprinting a full-size model of the human heart. ACS Biomater. Sci. Eng..

[197] Kačarević Z.P., Rider P., Alkildani S., Retnasingh S., Smeets R., Jung O., Ivanišević Z., Barbeck M. (2018). An introduction to 3D bioprinting: Possibilities, challenges and future aspects. Materials.

[198] Fedorovich N.E., Alblas J., De Wijn J.R., Hennink W.E., Verbout A.J., Dhert W.J. (2007). Hydrogels as extracellular matrices for skeletal tissue engineering: State-of-the-Art and novel application in organ printing. Tissue Eng..

[199] Guvendiren M., Burdick J.A. (2013). Engineering synthetic hydrogel microenvironments to instruct stem cells. Curr. Opin. Biotechnol..

[200] Dzobo K., Motaung K.S.C.M., Adesida A. (2019). Recent trends in decellularized extracellular matrix bioinks for 3D printing: An updated review. Int. J. Mol. Sci..

[201] Kabirian F., Mozafari M. (2020). Decellularized ECM-derived bioinks: Prospects for the future. Methods.

[202] Nam S.Y., Park S.-H., Noh I. (2018). ECM-Based Bioink for Tissue Mimetic 3D Printing, in Biomimetic Medical Materials: From Nanotechnology to 3D Printing.

[203] Rickard D., Sullivan T., Shenker B., Leboy P., Kazhdan I. (1994). Induction of rapid osteoblast differentiation in rat bone marrow stromal cell cultures by dexamethasone and BMP-2. Dev. Biol..

[204] Poldervaart M.T., Wang H., Van Der Stok J., Weinans H., Leeuwenburgh S.C.G., Öner F.C., Dhert W.J.A., Alblas J. (2013). Sustained release of BMP-2 in bioprinted alginate for osteogenicity in mice and rats. PLoS ONE.

[205] Balasundaram G., Sato M., Webster T.J. (2006). Using hydroxyapatite nanoparticles and decreased crystallinity to promote osteoblast adhesion similar to functionalizing with RGD. Biomaterials.

[206] Kim S.-S., Park M.S., Jeon O., Choi C.Y., Kim B.-S. (2006). Poly(lactide-co-glycolide)/hydroxyapatite composite scaffolds for bone tissue engineering. Biomaterials.

[207] Zhou X., Zhu W., Nowicki M., Miao S., Cui H., Holmes B., Glazer R.I., Zhang L.G. (2016). 3D bioprinting a cell-laden bone matrix for breast cancer metastasis study. ACS Appl. Mater. Interfaces.

[208] Jakus A.E., Rutz A.L., Jordan S.W., Kannan A., Mitchell S.M., Yun C., Koube K.D., Yoo S.C., Whiteley H.E., Richter C.-P. (2016). Hyperelastic “bone”: A highly versatile, growth factor–free, osteoregenerative, scalable, and surgically friendly biomaterial. Sci. Transl. Med..

[209] Huang Y.-H., Jakus A.E., Jordan S.W., Dumanian Z., Parker K., Zhao L., Patel P.K., Shah R.N. (2019). Three-Dimensionally printed hyperelastic bone scaffolds accelerate bone regeneration in critical-size calvarial bone defects. Plast. Reconstr. Surg..

[210] Alluri R., Jakus A., Bougioukli S., Pannell W., Sugiyama O., Tang A., Shah R., Lieberman J.R. (2018). 3D printed hyperelastic “bone” scaffolds and regional gene therapy: A novel approach to bone healing. J. Biomed. Mater. Res. Part A.

[211] Plantz M.A., Hsu W.K. (2020). Recent research advances in biologic bone graft materials for spine surgery. Curr. Rev. Musculoskelet. Med..

[212] Lewicki J., Bergman J., Kerins C., Hermanson O. (2019). Optimization of 3D bioprinting of human neuroblastoma cells using sodium alginate hydrogel. Bioprinting.

[213] Ferreira L.P., Gaspar V.M., Mano J.F. (2020). Decellularized extracellular matrix for bioengineering physiomimetic 3D in vitro tumor models. Trends Biotechnol..

[214] Zhao Y., Yao R., Ouyang L., Ding H., Zhang T., Zhang K., Cheng S., Sun W. (2014). Three-Dimensional printing of Hela cells for cervical tumor model in vitro. Biofabrication.

[215] Zhao S., Zuo W.-J., Shao Z.-M., Jiang Y.-Z. (2020). Molecular subtypes and precision treatment of triple-negative breast cancer. Ann. Transl. Med..

[216] Tang M., Xie Q., Gimple R.C., Prager B.C., Qiu Z., Schimelman J., Wang P., Lee D., Yu A., Miller T.E. (2020). Abstract 320: 3D-bioprinting of biomimetic multicellular glioblastoma tissues enable modeling of tumor-immune interactions. Mol. Cell. Biol. Genet..

[217] Hakobyan D., Médina C., Dusserre N., Stachowicz M.L., Handschin C., Fricain J.C., Guillermet-Guibert J., Oliveira H. (2020). Laser-Assisted 3D bioprinting of exocrine pancreas spheroid models for cancer initiation study. Biofabrication.

[218] Radhakrishnan J., Varadaraj S., Dash S.K., Sharma A., Verma R.S. (2020). Organotypic cancer tissue models for drug screening: 3D constructs, bioprinting and microfluidic chips. Drug Discov. Today.

[219] Zhu W., Castro N.J., Cui H., Zhou X., Boualam B., McGrane R., Glazer R.I., Zhang L.G. (2016). A 3D printed nano bone matrix for characterization of breast cancer cell and osteoblast interactions. Nanotechnology.

[220] Kolb A.D., Bussard K.M. (2019). The bone extracellular matrix as an ideal milieu for cancer cell metastases. Cancers.

[221] Ryan D.G., Murphy M.P., Frezza C., Prag H.A., Chouchani E.T., O’Neill L.A., Mills E.L. (2019). Coupling Krebs cycle metabolites to signalling in immunity and cancer. Nat. Metab..

[222] Bahraminasab M. (2020). Challenges on optimization of 3D-printed bone scaffolds. Biomed. Eng. Online.

[223] Wang L., Xu M., Luo L., Zhou Y., Si P. (2018). Iterative feedback bio-printing-derived cell-laden hydrogel scaffolds with optimal geometrical fidelity and cellular controllability. Sci. Rep..

[224] Wu Y., Heikal L., Ferns G.A., Ghezzi P., Nokhodchi A., Maniruzzaman M. (2019). 3D bioprinting of novel biocompatible scaffolds for endothelial cell repair. Polymers.

[225] Koons G.L., Mikos A.G. (2019). Progress in three-dimensional printing with growth factors. J. Control. Release.

[226] Foresti R., Rossi S., Pinelli S., Alinovi R., Barozzi M., Sciancalepore C., Galetti M., Caffarra C., Lagonegro P., Scavia G. (2020). Highly-Defined bioprinting of long-term vascularized scaffolds with Bio-Trap: Complex geometry functionalization and process parameters with computer aided tissue engineering. Materialia.

[227] Hanumantharao S.N., Que C.A., Vogl B.J., Rao S. (2020). Engineered Three-Dimensional Scaffolds Modulating Fate of Breast Cancer Cells Using Stiffness and Morphology Related Cell Adhesion. IEEE Open J. Eng. Med. Biol..

[228] Freeman F.E., Pitacco P., Van Dommelen L.H.A., Nulty J., Browe D.C., Shin J.-Y., Alsberg E., Kelly D.J. (2020). 3D bioprinting spatiotemporally defined patterns of growth factors to tightly control tissue regeneration. Sci. Adv..

[229] Sun B., Lian M., Han Y., Mo X., Jiang W., Qiao Z., Dai K. (2021). A 3D-Bioprinted dual growth factor-releasing intervertebral disc scaffold induces nucleus pulposus and annulus fibrosus reconstruction. Bioact. Mater..

